# Supplementary material for: Evaluation of Astatine-211-Labeled Fibroblast Activation Protein Inhibitor (FAPI): Comparison of Different Linkers with Polyethylene Glycol and Piperazine
Source: Int J Mol Sci. 2023 May 12;24(10):8701. doi: 10.3390/ijms24108701 (PMC10218645; doi:10.3390/ijms24108701)
Supplement: Supplementary file 1 [file ijms-24-08701-s001.zip › ijms-2345109-supplementary S1.pdf]

International Journal of
Molecular Sciences

MDPI

Evaluation of Astatine-211-Labeled Fibroblast Activation Protein Inhibitor (FAPI): Comparison of Different Linkers with Polyethylene Glycol and Piperazine

Ayaka Aso, Hinako Nabetani, Yoshifumi Matsuura, Yuichiro Kadonaga, Yoshifumi Shirakami, Tadashi Watabe*, Taku Yoshiya, Masayoshi Mochizuki, Kazuhiro Ooe, Atsuko Kawakami, Naoya Jinno, Atsushi Toyoshima, Hiromitsu Haba, Yang Wang, Jens Cardinale, Frederik L. Giesel, Atsushi Shimoyama, Kazuko Kaneda-Nakashima*, Koichi Fukase

Supporting information

Contents

1. Experimental section of chemical experiments

1-1. General method

For non-radiolabeled compounds

NMR spectra were recorded on the JEOL ECA500 spectrometer (500 MHz for ^1H and 126 MHz for ^{13}C) and Bruker AVANCENEO700 spectrometer (700 MHz for ^1H and 176 MHz for ^{13}C). Chemical shifts were reported in parts per million (ppm) relative to the internal residual solvent (^1H NMR, $CDCl_3$ 7.26 ppm, $(CD_3)_2SO$ 2.50 ppm, CD_3OD 3.31 ppm, D_2O 4.47 ppm; ^{13}C NMR, $CDCl_3$ 77.2 ppm, $(CD_3)_2SO$ 39.5 ppm, CD_3OD 49.0 ppm). ^{13}C NMR spectra with D_2O were measured with a small amount of CH_3CN (1.47 ppm) as an internal standard. Multiplicities are reported using the following abbreviations: s = singlet, d = doublet, t = triplet, q = quartet, m = multiplet, br = broad. High-resolution mass spectra were recorded on Thermo Fisher Scientific Q-Exactive Orbitrap (ESI-q-Orbitrap). Preparative HPLC was performed by LC-8A (Shimadzu), LC-20AP (Shimadzu) and LC-20AD (Shimadzu). For preparative HPLC of **S3**, **S5**, B-FAPI1 and B-FAPI2, YMC-ODS AA12305-2530WT (250 × 30 mm, YMC) was used. For preparative HPLC of B-FAPI3 and B-FAPI4, COSMOSIL $5C_{18}$-AR-300 (10 mm × 250 mm, Nacalai tesque) was used. All other commercially available reagents and solvents were used as purchased. Optical rotations were recorded on the Perkin-Elmer 241 Polarimeter.

For radiolabeled compounds

Radioactivity of ^{211}At was determined by γ-ray spectrometry with a Ge semiconductor detector (BE-2020, Mirion Technologies (Canberra) Inc.), Curie meter IGC-7 (Hitachi Ltd.), and Curie meter IGC-8B (Hitachi Ltd.). TLC silica gel 60 F_{254} (Merck) was used for TLC analysis of radiolabeled products. The TLC plate was exposed to an imaging plate (GE Healthcare), and the imaging plate was scanned by Typhoon FLA 7000 (GE Healthcare). Oasis HLB Plus Light Cartridge (weight: 30 mg, particle size: 30 μm, Waters corporation) was used for purification of ^{211}At-FAPI1, ^{211}At-FAPI2, ^{211}At-FAPI3, ^{211}At-FAPI4 and ^{211}At-FAPI5.

1-2. Abbreviation list

AcOH: Acetic acid
AcOEt: Ethyl acetate
DIPEA: *N*,*N*-Diisopropylethylamine
DMF: *N*,*N*-Dimethylformamide
EDC: 1-Ethyl-3-(3-dimethylaminopropyl)-carbodiimide
HATU: 1-[Bis(dimethylamino)methylene]-1H-1,2,3-triazolo[4,5-b]pyridinium 3-Oxide Hexafluorophosphate
HOAt: 1-Hydroxy-7-azabenzotriazole
MS4A: Molecular sieves, 4 Å
NMP: *N*-methylpyrrolidone
TMSOTf: Trimethylsilyl Trifluoromethanesulfonate
TFA: Trifluoroacetic acid

1-3. Synthetic procedure of B-FAPI1

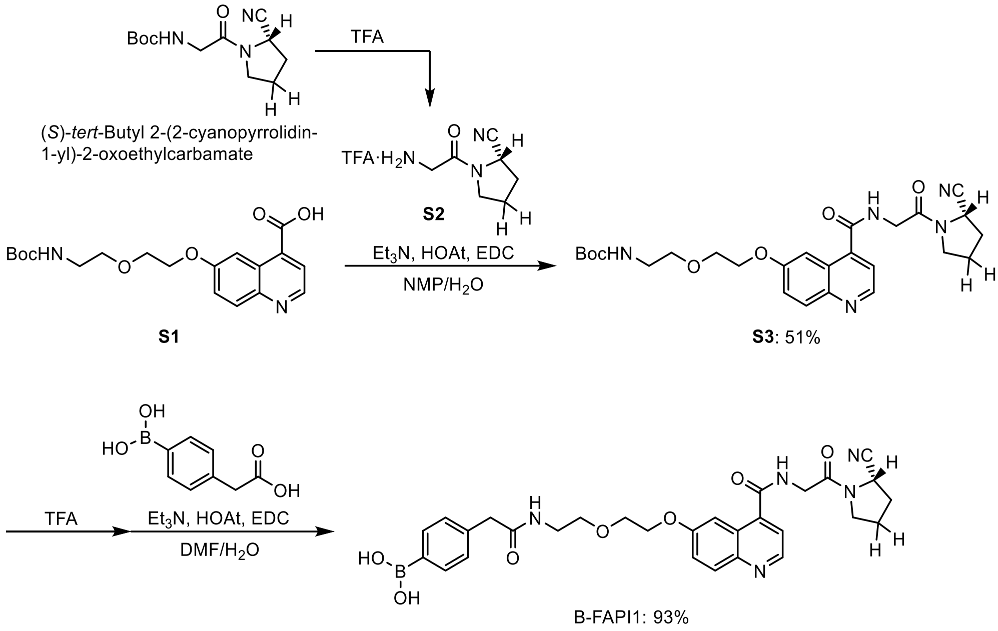

Scheme S1. Synthesis of B-FAPI1

Coupled ***S3***

A solution of (S)-tert-Butyl 2-(2-cyanopyrrolidin-1-yl)-2-oxoethylcarbamate (0.44 g, 1.74 mmol) in TFA (20 mL) was stirred for 30 minutes at 0 °C. The reaction mixture was concentrated to give amine S2, which was used for the next reaction without further purification.

To a solution of **S2** neutralized by Et_3N in NMP (10 mL) were added **S1** (0.62 g, 1.65 mmol) as a known compound,[1] HOAt (0.24 g, 1.76 mmol) and H_2O (1 mL) at 0 °C, and then EDC (0.6 mL, 3.3 mmol) was added to the mixture at 0 °C. After being stirred at room temperature for 3.5 h, the reaction mixture was diluted with AcOEt, washed with brine and concentrated. The residue was purified by reversed-phase HPLC (10 % to 60 % linear gradient of 0.05 % TFA in acetonitrile in 0.05 % TFA in water, over 80 min, flow rate of 20 mL/minute) to give coupled **S3** (0.43 g, 51 %): white solid; $[\alpha]_D^{22}$ -78 (*c* 0.1, CH_3OH); ^{1}H-NMR (500 MHz, $(CD_3)_2SO$) δ: 9.07 (t, *J* = 5.9 Hz, 1H), 8.91-8.86 (m, 1H), 8.03 (d, *J* = 9.2 Hz, 1H), 7.97 (s, 1H), 7.64-7.59 (m, 1H), 7.58-7.51 (m, 1H), 6.76 (brs, 1H), 4.82 (dd, *J* = 7.9, 3.5 Hz, 1H), 4.36-4.13 (m, 4H), 3.82 (t, *J* = 4.4 Hz, 2H), 3.74 (ddd, *J* = 9.5, 7.5, 4.2 Hz, 1H), 3.56 (dd, *J* = 16.0, 8.7 Hz, 1H), 3.47 (t, *J* = 6.1 Hz, 2H), 3.10 (q, *J* = 6.0 Hz, 2H), 2.37-1.89 (m, 4H), 1.36 (s, 9H); ^{13}C-NMR (126 MHz, $(CD_3)_2SO$) δ: 167.5, 167.0, 157.3, 155.6, 146.6, 142.5, 129.3, 125.7, 123.5, 119.3, 119.2, 104.8, 77.6, 69.3, 68.4, 67.9, 46.3, 45.3, 41.5, 29.4, 28.2, 24.9; HRMS (ESI-q-orbitrap) calcd for $C_{26}H_{33}N_5O_6Na$ $(M + Na)^+$ 534.2323, found: 534.2326.

B-FAPI1

A solution of **S3** (0.23 g, 0.45 mmol) in TFA (5.0 mL) was stirred at 0 °C for 30 minutes. After the reaction mixture was concentrated, the residues were dissolved in DMF (2 mL) and neutralized with Et_3N. To a mixture were added 4-(Carboxymethyl)phenylboronic acid (89 mg, 0.50 mmol), HOAt (67 mg, 0.50 mmol) and H_2O (0.2 mL) at room temperature, and then EDC (0.11 mL, 0.63 mmol) was added to the mixture at 0 °C. After being stirred at room temperature for 1 h, the reaction mixture was diluted with TFA/H_2O (0.1:99.9). Reversed phase HPLC (10 % to 50 % linear gradient of 0.1 % TFA in acetonitrile in 0.1 % TFA in water, over 80 min, flow rate of 20 mL/min) gave B-FAPI1 (0.24 g, 93 %): white solid; $[\alpha]_D^{22}$ -22 (*c* 0.1, CH_3CN); ^{1}H-NMR (700 MHz, $(CD_3)_2SO$) δ: 9.09 (t, *J* = 6.0 Hz,

1H), 8.90 (d, *J* = 4.5 Hz, 1H), 8.10 (t, *J* = 5.6 Hz, 1H), 8.04 (d, *J* = 9.2 Hz, 1H), 7.98 (d, *J* = 2.7 Hz, 1H), 7.69 (d, *J* = 8.0 Hz, 2H), 7.62 (d, *J* = 4.6 Hz, 1H), 7.55 (dd, *J* = 9.1, 2.8 Hz, 1H), 7.20 (d, *J* = 7.8 Hz, 2H), 4.81 (dd, *J* = 7.9, 3.5 Hz, 1H), 4.35-4.28 (m, 2H), 4.25 (dd, *J* = 16.8, 6.2 Hz, 1H), 4.21 (dd, *J* = 16.8, 5.8 Hz, 1H), 3.83 (t, *J* = 4.4 Hz, 2H), 3.73 (ddd, *J* = 9.4, 7.6, 4.1 Hz, 1H), 3.55 (dd, *J* = 16.0, 8.6 Hz, 1H), 3.51 (t, *J* = 5.9 Hz, 2H), 3.40 (s, 2H), 3.28-3.21 (m, 2H), 2.27-2.13 (m, 2H), 2.12-2.00 (m, 2H); ^{13}C-NMR (176 MHz, $(CD_3)_2SO$) δ: 170.1, 167.4, 167.0, 157.3, 146.4, 142.7, 142.1, 138.3, 134.0, 129.1, 128.0, 125.8, 123.6, 119.3, 119.2, 104.8, 69.2, 68.5, 67.8, 46.3, 45.3, 42.4, 41.5, 40.0, 38.7, 29.4, 24.8; HRMS (ESI-q-orbitrap) calcd for $C_{29}H_{32}BN_5O_7Na$ $(M + Na)^+$ 596.2287, found: 596.2294.

1-4. Synthetic procedure of B-FAPI2

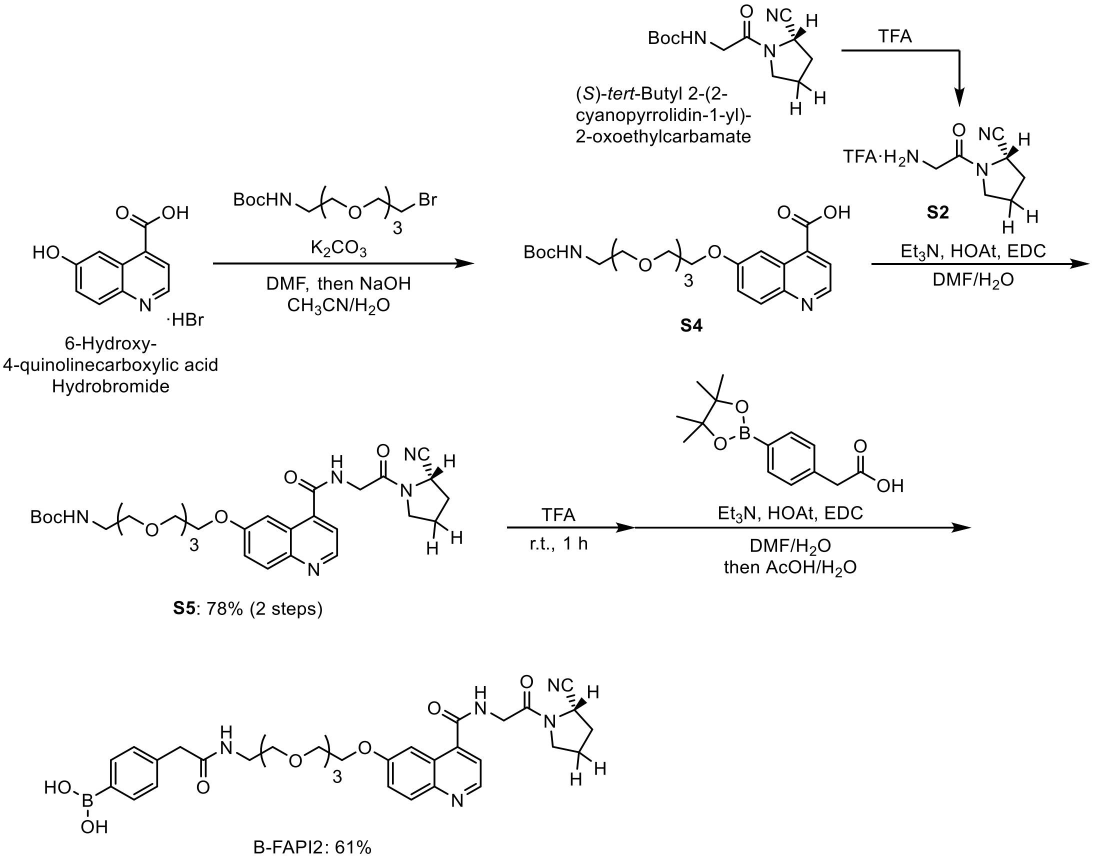

Scheme S2. Synthesis of B-FAPI2

Coupled **S5**

K_2CO_3 (1.6 g, 12 mmol) and tert-Butyl (2-(2-(2-(2-bromoethoxy)ethoxy)ethoxy)ethyl)carbamate (1.3 g, 3.6 mmol) were added to a solution of 6-Hydroxy-4-quinolinecarboxylic acid hydrobromide (0.32 g, 1.2 mmol) in DMF (5 mL) at 65 °C. After being stirred at 65 °C for 4 h, the reaction mixture was cooled down to room temperature, filtered through filter paper and concentrated. The residues were dissolved in CH_3CN/H_2O (3:2) (10 mL) and 5 M NaOH in H_2O (1.2 mL, 6.0 mmol) was added at 0 °C and the reaction mixture was stirred at room temperature for 30 minutes. After the reaction was quenched with AcOH at 0 °C, the mixture was concentrated. The residue was dissolved in AcOEt. The organic layer was washed with brine, dried over Na_2SO_4 and concentrated. The residue was crystalized with $CHCl_3$ and Hexane to give *N*-Boc-PEG4 **S4**, which was used for the next reaction without further purification.

A solution of (*S*)-tert-Butyl 2-(2-cyanopyrrolidin-1-yl)-2-oxoethylcarbamate (301 mg, 1.19 mmol) in TFA (10 mL) was stirred at 0 °C for 40 minutes. The reaction mixture was concentrated to give amine **S2**, which was used for the next reaction without further purification.

S4, HOAt (0.18 g, 1.3 mmol) and H_2O (1 mL) were added to a solution of **S2** neutralized by Et_3N in DMF (9 mL) at room temperature. After the mixture was cooled down to 0 °C, EDC (0.24 mL, 1.3 mmol) was added and the reaction mixture was stirred at room temperature for 1.5 h, and then concentrated. The residue was purified by reversed phase HPLC (10 % to 60 % linear gradient of 0.05 % TFA in acetonitrile in 0.05 % TFA in water, over 80 min, flow rate of 20 mL/minute) to give coupled **S5** (0.56 g, 78 % in 2 steps): white solid; $[\alpha]_D^{22}$ -51 (*c* 0.1, CH_3OH); ^{1}H-NMR (500 MHz, $(CD_3)_2SO$) δ: 9.06 (t, *J* = 6.0 Hz, 1H), 8.87 (d, *J* = 4.5 Hz, 1H), 8.02 (d, *J* = 9.2 Hz, 1H), 7.96 (d, *J* = 2.8 Hz, 1H), 7.59 (d, *J* = 4.6 Hz, 1H), 7.54 (dd, *J* = 9.2, 2.8 Hz, 1H), 6.71 (s, 1H), 4.82 (dd, *J* = 7.9, 3.5 Hz, 1H), 4.33-4.25 (m, 2H), 4.29-4.16 (m, 2H), 3.84 (t, *J* = 4.5 Hz, 2H), 3.77-3.70 (m, 1H), 3.64-3.58 (m, 2H), 3.59-3.52 (m, 3H), 3.54-3.45 (m, 4H), 3.37 (t, *J* = 6.1 Hz, 2H), 3.05 (q, *J* = 5.9 Hz, 2H), 2.29-2.12 (m, 2H), 2.15-2.00 (m, 2H), 1.36 (s, 9H); ^{13}C-NMR ($(CD_3)_2SO$) δ: 167.5, 167.4, 156.9, 155.6, 147.5, 144.1, 141.0, 130.7, 125.4, 122.5, 119.2, 119.1, 104.6, 77.6, 69.9, 69.7, 69.7, 69.5, 69.1, 68.7, 67.7, 46.3, 45.3, 41.5, 29.4, 28.2, 24.9.; HRMS (ESI-q-orbitrap) calcd for $C_{30}H_{41}N_5O_8Na$ $(M + Na)^+$ 622.2847, found: 622.2853.

B-FAPI2

A solution of **S5** (0.21 g, 0.35 mmol) in TFA (5 mL) was stirred at room temperature for 1 h. After the reaction mixture was concentrated, the residue was dissolved in DMF (2 mL) and neutralized with Et_3N. To the neutralized mixture were added 4-(Carboxymethyl)phenylboronic acid pinacol ester (0.10 g, 0.38 mmol), HOAt (52 mg, 0.39 mmol) and H_2O (0.2 mL) at room temperature. After the mixture was cooled down to -78 °C, EDC (69 μL, 0.39 mmol) was added and the reaction mixture was stirred for 1.5 h at room temperature, and then concentrated. The residue was dissolved in AcOH/H_2O (1:4) (2 mL). After being stirred at room temperature for 17 h, the reaction mixture was diluted with H_2O. Reversed-phase HPLC (10 % to 50 % linear gradient of 0.1 % TFA in acetonitrile in 0.1 % TFA in water, over 80 minutes, flow rate of 20 mL/minute) gave B-FAPI2 (0.14 g, 61%): white solid; $[\alpha]_D^{22}$ -17 (*c* 0.1, CH_3CN); ^{1}H-NMR (700 MHz, $(CD_3)_2SO$) δ: 9.07 (t, *J* = 6.0 Hz, 1H), 8.88 (d, *J* = 4.4 Hz, 1H), 8.05 (t, *J* = 5.7 Hz, 1H), 8.02 (d, *J* = 9.1 Hz, 1H), 7.97 (d, *J* = 2.8 Hz, 1H), 7.69 (d, *J* = 7.6 Hz, 2H), 7.60 (d, *J* = 4.6 Hz, 1H), 7.54 (dd, *J* = 9.2, 2.8 Hz, 1H), 7.20 (d, *J* = 7.6 Hz, 2H), 4.81 (dd, *J* = 8.0, 3.4 Hz, 1H), 4.33-4.27 (m, 2H), 4.25 (dd, *J* = 16.8, 6.2 Hz, 1H), 4.20 (dd, *J* = 16.8, 5.7 Hz, 1H), 3.84 (t, *J* = 4.4 Hz, 2H), 3.75-3.70 (m, 1H), 3.63-3.59 (m, 2H), 3.57-3.53 (m, 3H), 3.53-3.50 (m, 2H), 3.50-3.47 (m, 2H), 3.41-3.38 (m, 4H), 3.19 (q, *J* = 5.8 Hz, 2H), 2.25-2.19 (m, 1H), 2.19-2.13 (m, 1H), 2.12-2.00 (m, 2H); ^{13}C-NMR (176 MHz, $(CD_3)_2SO$) δ: 170.0, 176.5, 167.0, 157.2, 146.6, 142.4, 138.3, 134.0, 129.4, 128.0, 125.7, 123.4, 119.3, 119.2, 104.8, 69.9, 69.7, 69.7, 69.5, 69.0, 68.6, 67.8, 46.3, 45.3, 42.4, 41.5, 40.0, 38.7, 29.4, 24.9; HRMS (ESI-q-orbitrap) calcd for $C_{33}H_{40}BN_5O_9Na$ $(M + Na)^+$ 684.2811, found: 684.2819.

1-5. Synthetic procedure of B-FAPI3

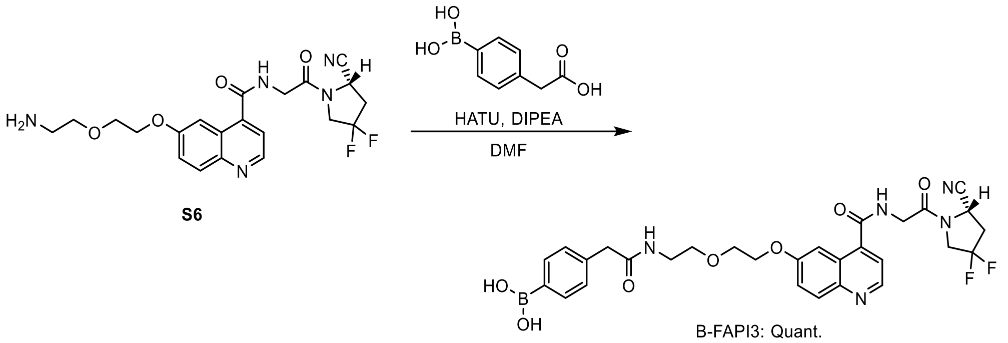

Scheme S3. Synthesis of B-FAPI3

B-FAPI3

DIPEA (20.0 μL, 115 μmol)was added to a solution of 4-(Carboxymethyl) phenylboronic acid (8.0 mg, 44.5 μmol) and HATU (14.0 mg, 36.8 μmol) in DMF (1.0 mL) , and the mixture was stirred at room temperature for 5 minutes. After **S6** (2.8 mg, 6.3 μmol) as a known compound[1] was added to the mixture, the resulting mixture was stirred at room temperature for 19.5 h. The reaction mixture was concentrated. The residue was purified by reversed phase HPLC (2 % to 98 % linear gradient of 0.1 % TFA in acetonitrile in water, over 40 minutes, flow rate of 7.0 mL/minute) to give B-FAPI3 (3.8 mg, quant.): white solid; $[\alpha]_D^{22}$ -9 (*c* 0.1, CH_3CN); ^{1}H-NMR (700 MHz, $(CD_3)_2SO$) δ: 9.06 (t, *J* = 6.0 Hz, 1H), 8.81 (d, *J* = 4.3 Hz, 1H), 7.99 (d, *J* = 9.2 Hz, 1H), 7.92 (s, 1H), 7.89 (d, *J* = 2.8 Hz, 1H), 7.69 (d, *J* = 7.8 Hz, 2H), 7.51 (d, *J* = 4.2 Hz, 1H), 7.47 (dd, *J* = 9.2, 2.9 Hz, 1H), 7.20 (d, *J* = 7.7 Hz, 2H), 5.14 (dd, *J* = 9.5, 2.8 Hz, 1H), 4.37-4.27 (m, 1H), 4.31-4.24 (m, 2H), 4.29-4.18 (m, 2H), 4.18-4.09 (m, 1H), 3.82 (t, *J* = 4.4 Hz, 2H), 3.50 (t, *J* = 5.8 Hz, 2H), 3.40 (s, 2H), 3.26-3.22 (m, 2H), 2.96-2.85 (m, 1H), 2.81 (t, *J* = 14.0 Hz, 1H); ^{13}C-NMR (176 MHz, $(CD_3)_2SO$) δ: 170.1, 170.0, 168.0, 167.5, 156.8, 147.5, 144.1, 140.8, 138.2, 134.0, 130.7, 128.0, 125.3, 122.5, 119.1, 117.7, 104.5, 69.2, 68.5, 67.6, 51.2, 44.2, 44.2, 42.3, 41.3, 40.0, 38.5, 36.3; HRMS (ESI-q-orbitrap) calcd for $C_{29}H_{30}BF_2N_5O_7Na$ $(M + Na)^+$ 632.2099, found: 632.2104.

1-6. Synthetic procedure of B-FAPI4

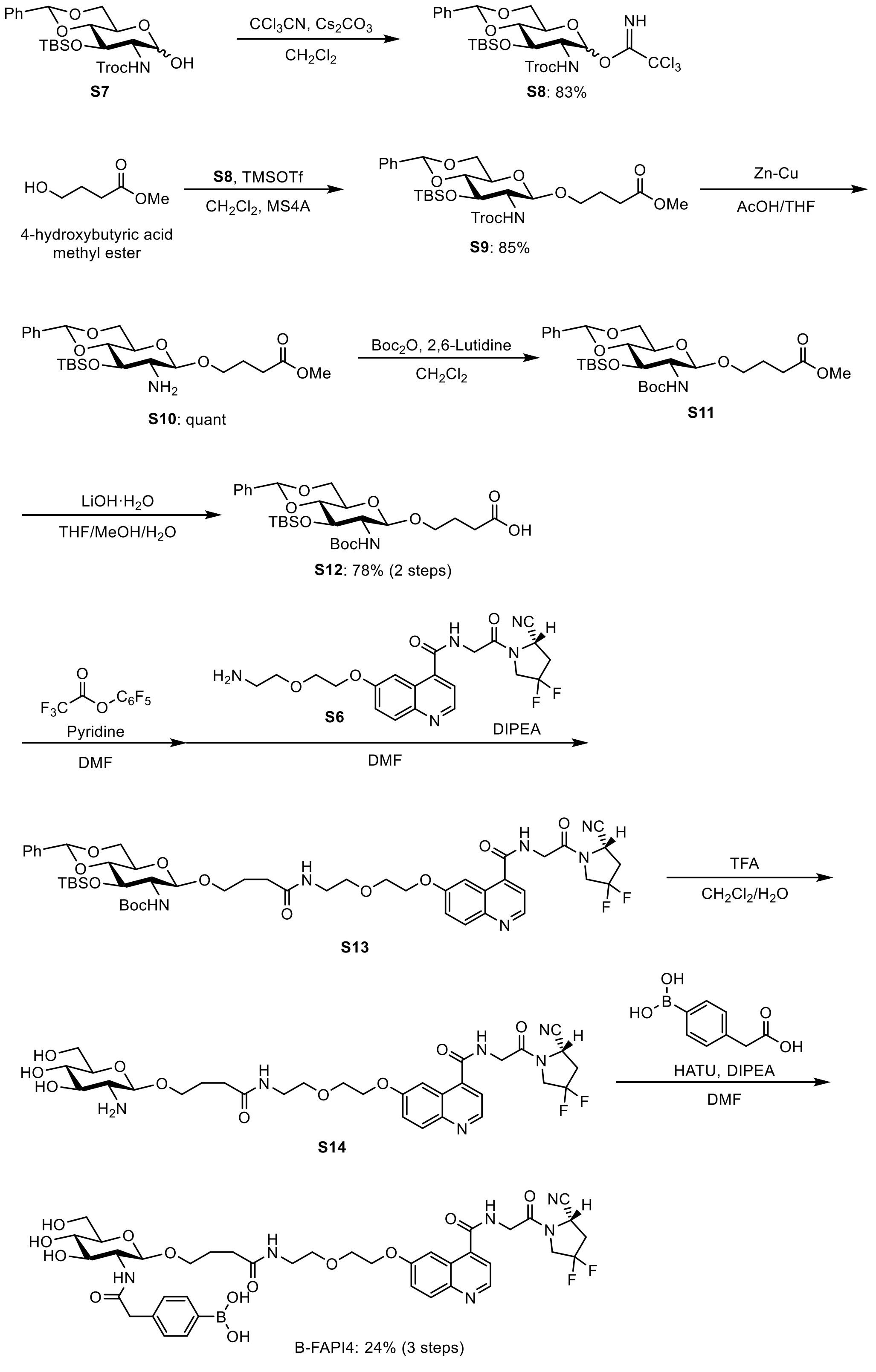

Scheme S4. Synthesis of B-FAPI4

*Imidate **S8***

CCl_3CN (6.6 mL, 65.8 mmol) and Cs_2CO_3 (5.38 g, 16.5 mmol) were added to a solution of **S7** (4.6 g, 8.3 mmol) as a known compound[2] in CH_2Cl_2 (160 mL) at room temperature. After being stirred at room temperature for 40 minutes, the reaction mixture was filtered through a Celite pad and washed with CH_2Cl_2, and then concentrated. The residue was purified by silica-gel column chromatography (Toluene/AcOEt, 30:1) to give imidate **S8** (4.8 g, 83 %): colorless amorphous oil; ^{1}H-NMR ($CDCl_3$, 500 MHz) δ: 8.74 (s, 1H), 7.53-7.45 (m, 2H), 7.40-7.33 (m, 3H), 6.33 (d, *J* = 3.8 Hz, 1H), 5.56 (s, 1H), 4.97 (d, *J* = 9.7 Hz, 1H), 4.79 (d, *J* = 12.0 Hz, 1H), 4.61 (d, *J* = 11.9 Hz, 1H), 4.32 (dd, *J* = 10.4, 4.9 Hz, 1H), 4.17 (td, *J* = 9.7, 3.8 Hz, 1H), 4.04-3.91 (m, 2H), 3.78 (t, *J* = 10.3 Hz, 1H), 3.66 (t, *J* = 9.3 Hz, 1H), 0.84 (s, 9H), 0.08 (s, 3H), 0.02 (s, 3H).

*Glycoside **S9***

4-Hydroxybutyric acid methyl ester (1.9 mL, 17.2 mmol) was added to a mixture of **S8** (4.0 g, 5.70 mmol) and MS4A in CH_2Cl_2 (115 mL) at room temperature. After the mixture was cooled down to 0 °C, TMSOTf (103 μL, 570 μmol) was added and the reaction mixture was stirred at the same temperature for 20 minutes, and then warmed up to room temperature. The reaction was quenched with Et_3N. The mixture was extracted with AcOEt. The organic layer was washed with H_2O and brine, dried over Na_2SO_4 and concentrated. The residue was purified via silica-gel column chromatography (Hexane/Acetone, 89:11) gave glycoside **S9** (3.2 g, 85 %): white solid; ^{1}H-NMR (500 MHz, $CDCl_3$) δ: 7.49-7.40 (m, 2H), 7.37-7.25 (m, 3H), 5.83-5.56 (m, 1H), 5.37 (s, 1H), 4.77-4.64 (m, 2H), 4.52 (d, *J* = 8.5 Hz, 1H), 4.26 (dd, *J* = 10.0, 4.3 Hz, 1H), 3.90 (t, *J* = 8.5 Hz, 1H), 3.90-3.80 (m, 1H), 3.70 (t, *J* = 10.1 Hz, 1H), 3.63 (s, 3H), 3.55-3.35 (m, 3H), 3.42-3.27 (m, 1H), 2.45-2.30 (m, 2H), 1.93-1.78 (m, 2H), 0.81 (s, 9H), 0.03 (s, 3H), -0.03 (s, 3H); ^{13}C-NMR (126 MHz, $CDCl_3$) δ: 173.8, 154.2, 137.2, 129.0, 128.0, 126.3, 101.5, 101.2, 95.4, 82.0, 74.6, 72.0, 68.5, 68.4, 65.9, 59.3, 51.5, 30.3, 25.7, 24.8, 18.1, -4.1, -4.9; HRMS (ESI-q-orbitrap) calcd for $C_{27}H_{40}Cl_3NO_9SiNa$ $(M + Na)^+$ 678.1430, found: 678.1433.

*Amine **S10***

Activated Zn-Cu (prepared from 1.5 g of Zn) was added to a solution of **S9** (500 mg, 761 μmol) in AcOH/THF (1:1) (76 mL) at room temperature. After being stirred at the same temperature for 2 h, the reaction mixture was filtered through a celite pad with AcOEt. The filtrate was concentrated with toluene, and then the residue was neutralized with saturated aqueous $NaHCO_3$. The mixture was extracted with AcOEt. The organic layer was washed with water and brine, dried over Na_2SO_4 and concentrated. The residue was purified via silica-gel column chromatography (Toluene/AcOEt, 5:1, 2:1) to give amine **S10** (367 mg, quant.): colorless oil; ^{1}H-NMR (500 MHz, $(CD_3)_2SO$) δ: 7.45-7.40 (m, 2H), 7.39-7.34 (m, 3H), 5.60 (s, 1H), 4.31 (d, *J* = 7.9 Hz, 1H), 4.17 (dd, *J* = 10.2, 4.8 Hz, 1H), 3.79-3.69 (m, 2H), 3.59 (s, 3H), 3.57 (t, *J* = 8.9 Hz, 2H), 3.52-3.37 (m, 3H), 2.39 (t, *J* = 7.4 Hz, 2H), 1.84-1.75 (m, 2H), 1.53 (s, 2H), 0.82 (s, 9H), 0.05 (s, 3H), -0.05 (s, 3H); ^{13}C-NMR (126 MHz, $(CD_3)_2SO$) δ: 173.1, 137.6, 128.8, 127.9, 126.1, 103. 7, 100.8, 81.1, 74.8, 67.9, 67.8, 65.8, 58.8, 51.2, 29.9, 25.8, 24.6, 18.0, -4.1, -4.7; HRMS (ESI-q-orbitrap) calcd for $C_{24}H_{40}NO_7Si$ $(M + H)^+$ 482.2569, found: 482.2566.

*Carboxylic acid **S12***

2,6-Lutidine (220 μL, 1.89 mmol) and Boc_2O (2.0 mL, 6.76 mmol) were added to a solution of **S10** (329 mg, 682 mmol) in CH_2Cl_2 (68 mL) at room temperature. After the reaction mixture was stirred at room temperature for 61 h, the reaction was quenched with sat. aqueous NH_4Cl. The mixture was extracted with CH_2Cl_2. The organic layer was washed with H_2O and brine, dried over Na_2SO_4 and concentrated. The residue was purified via silica-gel

column chromatography (Toluene/EtOAc, 100:0, 1:1) to give *N*-Boc **S11**, which was used for the next reaction without further purification.

LiOH·H_2O (172 mg, 4.10 mmol) was added to a solution of **S11** in THF/MeOH/H_2O (3:2:2) (70 mL) at 0 °C. After the reaction mixture was stirred at room temperature for 19 h, the reaction was quenched with 0.5 M aqueous HCl. The mixture was extracted with CH_2Cl_2. The organic layer was washed with H_2O and brine, dried over Na_2SO_4 and concentrated. The residue was purified via silica-gel column chromatography (Toluene/ AcOEt, 9:1) to give Carboxylic acid **S12** (304 mg, 78% in 2 steps): white solid; ^{1}H-NMR (500 MHz, CD_3OD) δ: 7.49-7.43 (m, 2H), 7.36-7.31 (m, 3H), 6.73 (d, *J* = 9.8 Hz, 1H), 5.51 (s, 1H), 4.43 (d, *J* = 8.5 Hz, 1H), 4.24 (dd, *J* = 10.3, 4.9 Hz, 1H), 3.87 (dt, *J* = 9.9, 5.8 Hz, 1H), 3.78 (t, *J* = 9.2 Hz, 1H), 3.76 (t, *J* = 10.1 Hz, 1H), 3.53 (dt, *J* = 10.0, 6.2 Hz, 1H), 3.45 (q, *J* = 9.8 Hz, 2H), 3.46-3.35 (m, 1H), 2.47-2.34 (m, 2H), 1.90-1.78 (m, 2H), 1.45 (s, 9H), 0.84 (s, 9H), 0.06 (s, 3H), -0.03 (s, 3H); ^{13}C-NMR (126 MHz, CD_3OD) δ: 177.2, 158.1, 139.1, 130.0, 129.0, 127.6, 103.8, 103.0, 83.6, 80.1, 74.2, 69.8, 69.7, 67.3, 59.6, 31.3, 28.9, 26.5, 26.1, 19.1, -3.9, -4.5; HRMS (ESI-q-orbitrap) calcd for $C_{28}H_{44}NO_9Si^-$ $(M - H)^-$ 566.2791, found: 566.2791.

B-FAPI4

Pyridine (7.1 μL, 88.1 μmol) and Pentafluorophenyl trifluoroacetate (15.0 μL, 88.1 μmol) were added to a solution of **S12** (10.0 mg, 17.6 μmol) in DMF (1.8 mL) at room temperature. After being stirred at room temperature for 2.5 h, the reaction mixture was concentrated and dried in vacuo. The residue was dissolved in DMF (3 mL) and **S6** (7.9 mg, 17.8 μmol) and DIPEA (14.7 μL, 84.5 μmol) were added to the mixture at room temperature. After being stirred at room temperature for 24 h, the reaction mixture was concentrated. The residue was purified by preparative thin layer chromatography with Acetone and CH_2Cl_2 to give coupled **S13**, which was used for the next reaction without further purification.

TFA (160 μL) was added to a solution of **S13** in CH_2Cl_2/H_2O (10:1) (1.76 mL) at 0 °C. After being stirred at room temperature for 2.5 h, the reaction mixture was concentrated. The residue was purified by reversed phase HPLC (2 % to 98 % linear gradient of acetonitrile in 0.1% TFA in water, over 60 min, flow rate of 7.0 mL/minute) to give **S14**, which was used for the next reaction without further purification.

DIPEA (2.4 μL, 13.5 μmol) was added to a solution of 4-(Carboxymethyl)phenylboronic acid (2.0 mg, 11.2 μmol) and HATU (4.3 mg, 11.2 μmol) in DMF (0.5 mL) . The mixture was stirred at room temperature for 5 minutes. Then, **S14** in DMF was added. The mixture was stirred at room temperature for 4 h. After distilling the reaction solution under reduced pressure, the residue was purified by reversed phase HPLC (2 % to 98 % linear gradient of acetonitrile in 0.1% TFA in water, over 53 min, then 2 % to 98 % linear gradient of acetonitrile in 0.1% TFA in water, over 40 minutes, flow rate of 7.0 mL/minute) to give B-FAPI4 (3.6 mg, 24 % in 3 steps): white solid; $[\alpha]_D^{22}$ -1 (*c* 0.1, CH_3CN); ^{1}H-NMR (700 MHz, D_2O) δ: 8.85 (d, *J* = 4.6 Hz, 1H), 8.08-8.00 (m, 1H), 7.71 (d, *J* = 4.5 Hz, 1H), 7.65 (d, *J* = 2.7 Hz, 1H), 7.62 (d, *J* = 7.4 Hz, 2H), 7.56 (dd, *J* = 9.2, 2.8 Hz, 2H), 7.26 (d, *J* = 7.6 Hz, 2H), 5.22 (dd, *J* = 9.0, 3.9 Hz, 1H), 4.46-4.31 (m, 6H) , 4.28-4.18 (m, 1H), 4.02 (t, *J* = 4.2 Hz, 2H), 3.93 (d, *J* = 12.2 Hz, 1H), 3.79 (t, *J* = 5.3 Hz, 2H), 3.78-3.64 (m, 3H), 3.62-3.53 (m, 3H), 3.50-3.39 (m, 4H), 3.36-3.30 (m, 1H), 3.09-2.96 (m, 1H), 2.00-1.84 (m, 2H), 1.60-1.54 (m, 2H); ^{13}C-NMR (700 MHz, D_2O) δ: 176.7, 169.8, 163.5, 158.0, 134.6, 129.1, 104.7, 101.5, 76.4, 74.2, 70.6, 69.7, 69.5, 69.3, 68.4, 61.4, 56.2, 45.4, 43.5, 42.7, 39.7, 32.9, 30.9, 25.9; HRMS (ESI-q-orbitrap) calcd for $C_{39}H_{47}BF_2N_6O_{13}Na$ $(M + Na)^+$ 879.3154, found: 879.3160.

1-7. Synthetic procedure of B-FAPI5

B-FAPI5 was synthesized according to a procedure laid out in the literature.[3]

1-8. ^{1}H- and ^{13}C NMR spectra

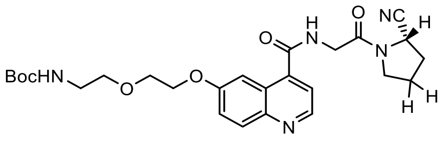

S3

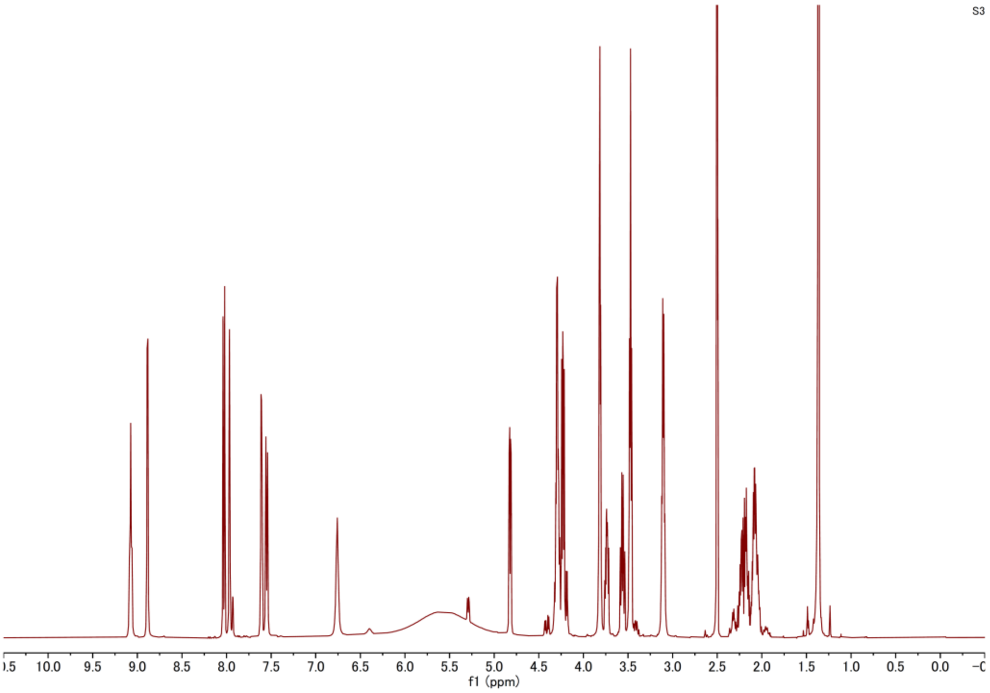

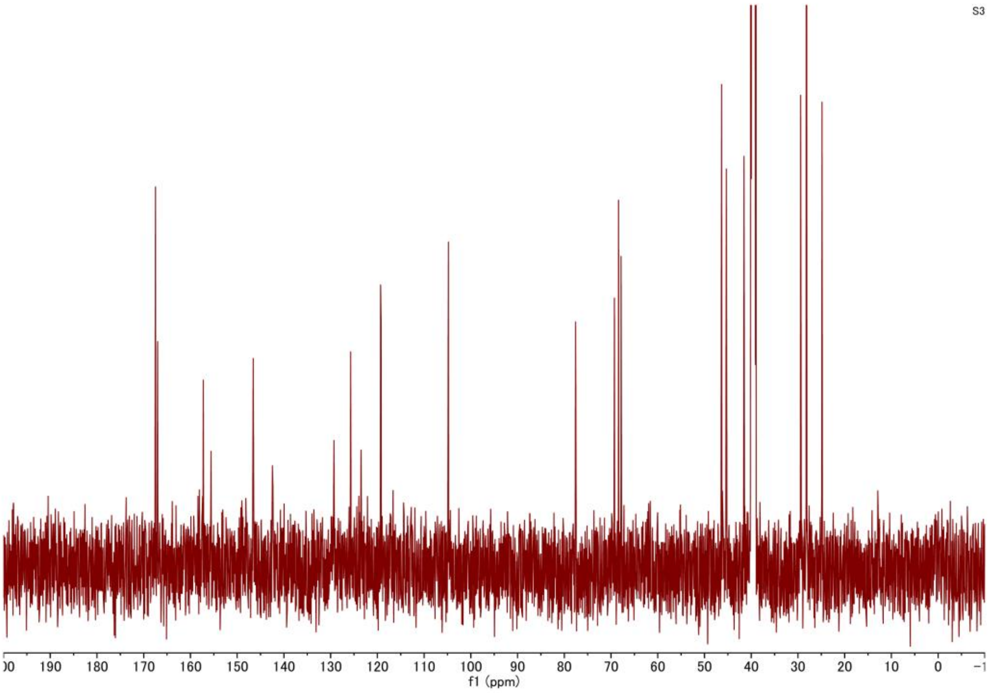

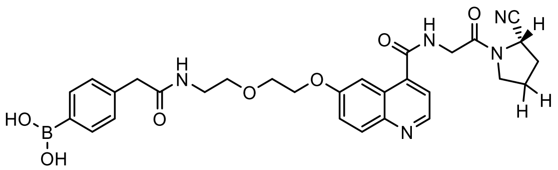
HO
B
OH
H
N
O
O
O
O
H
N
O
NC
H
N
N
H
H

B-FAPI1

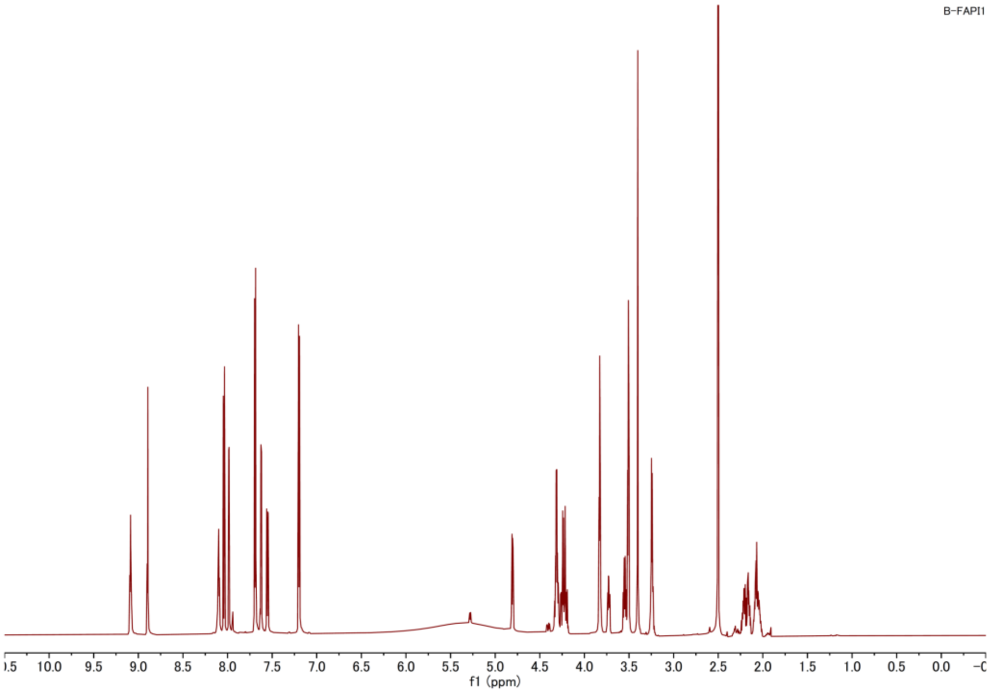
B-FAPI1
10.0 9.5 9.0 8.5 8.0 7.5 7.0 6.5 6.0 5.5 5.0 4.5 4.0 3.5 3.0 2.5 2.0 1.5 1.0 0.5 0.0
f1 (ppm)

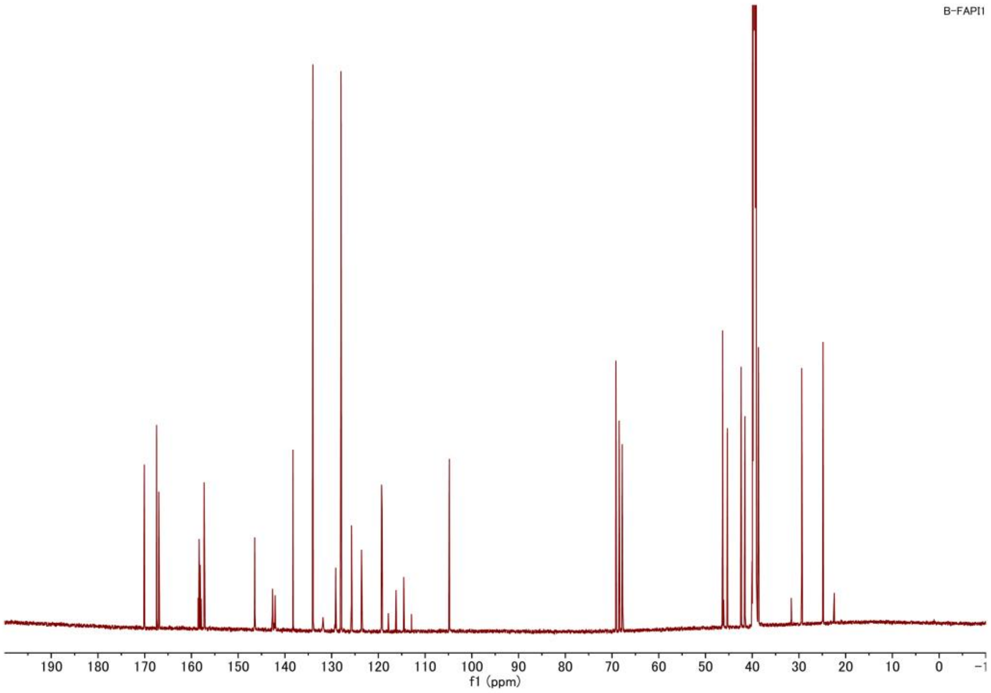
B-FAPI1
190 180 170 160 150 140 130 120 110 100 90 80 70 60 50 40 30 20 10 0
f1 (ppm)

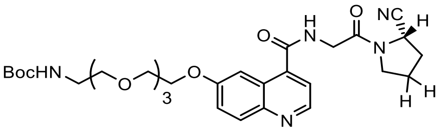
BocHN
O
3
O
O
H
N
O
NC
H
N
N
H
H

S5

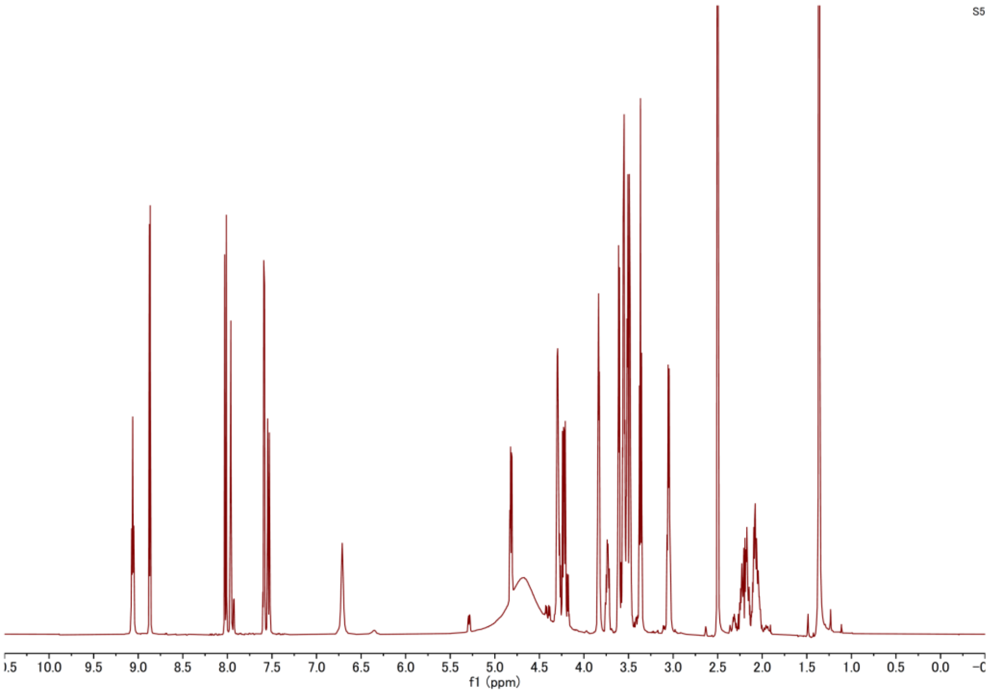
S5
10.0 9.5 9.0 8.5 8.0 7.5 7.0 6.5 6.0 5.5 5.0 4.5 4.0 3.5 3.0 2.5 2.0 1.5 1.0 0.5 0.0
f1 (ppm)

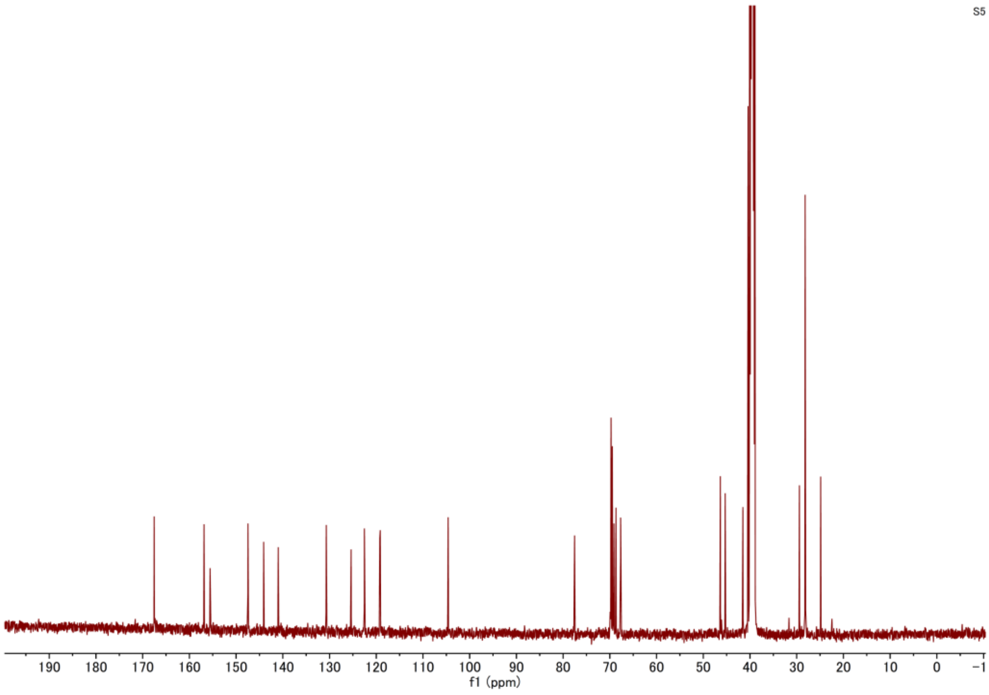
S5
190 180 170 160 150 140 130 120 110 100 90 80 70 60 50 40 30 20 10 0
f1 (ppm)

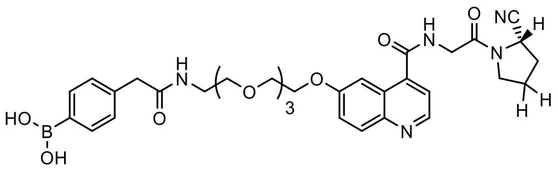

B-FAPI2

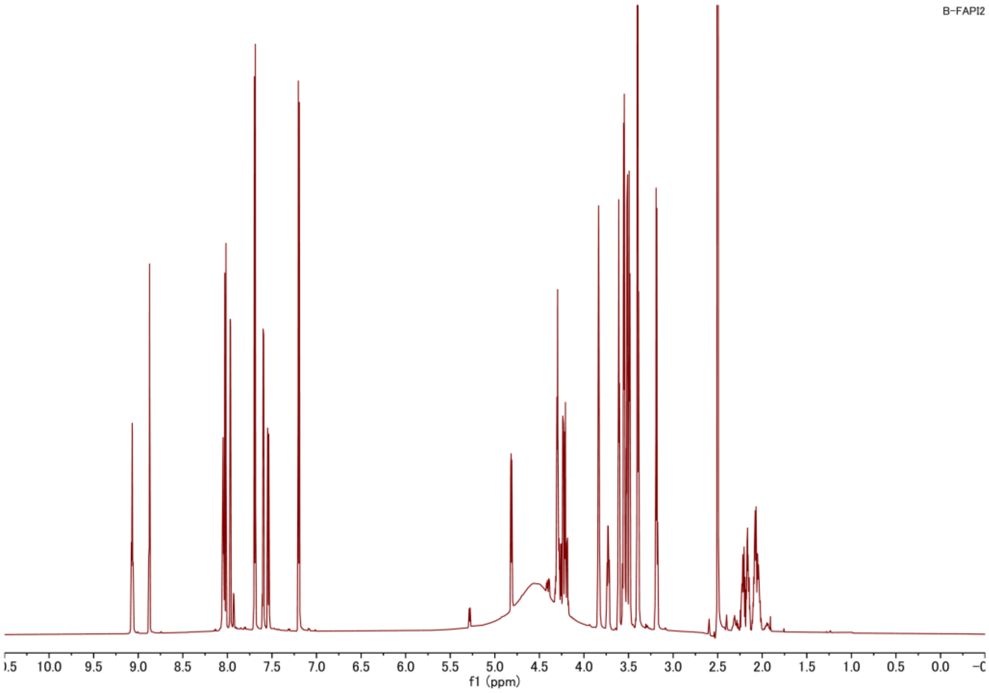

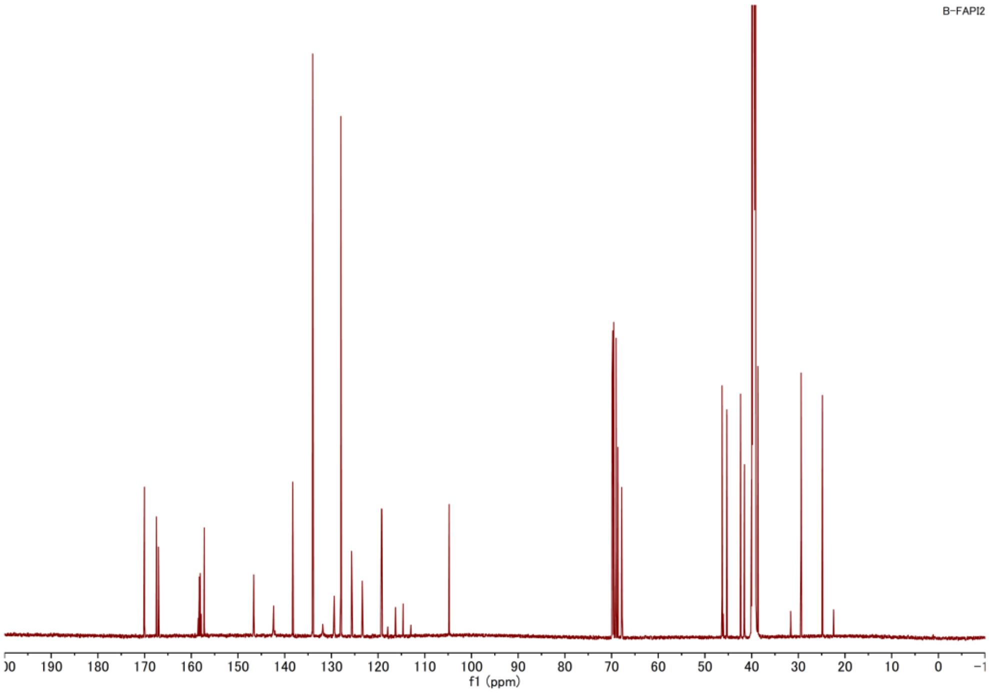

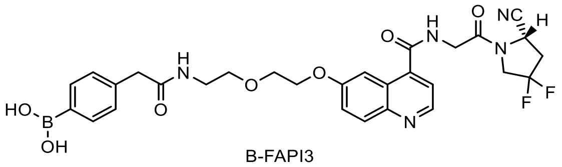

B-FAPI3

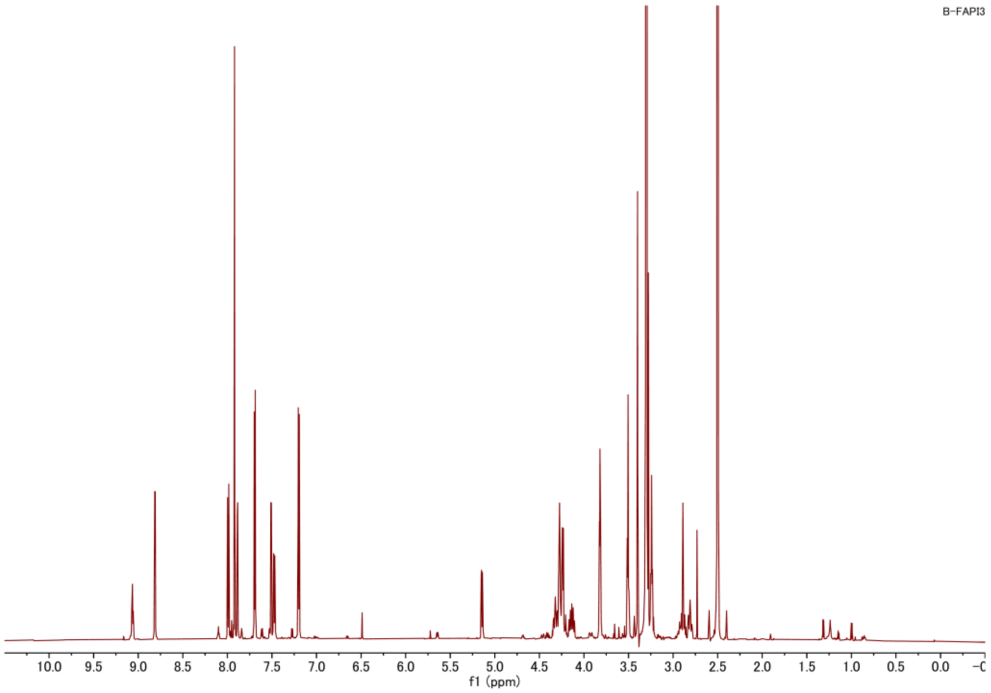

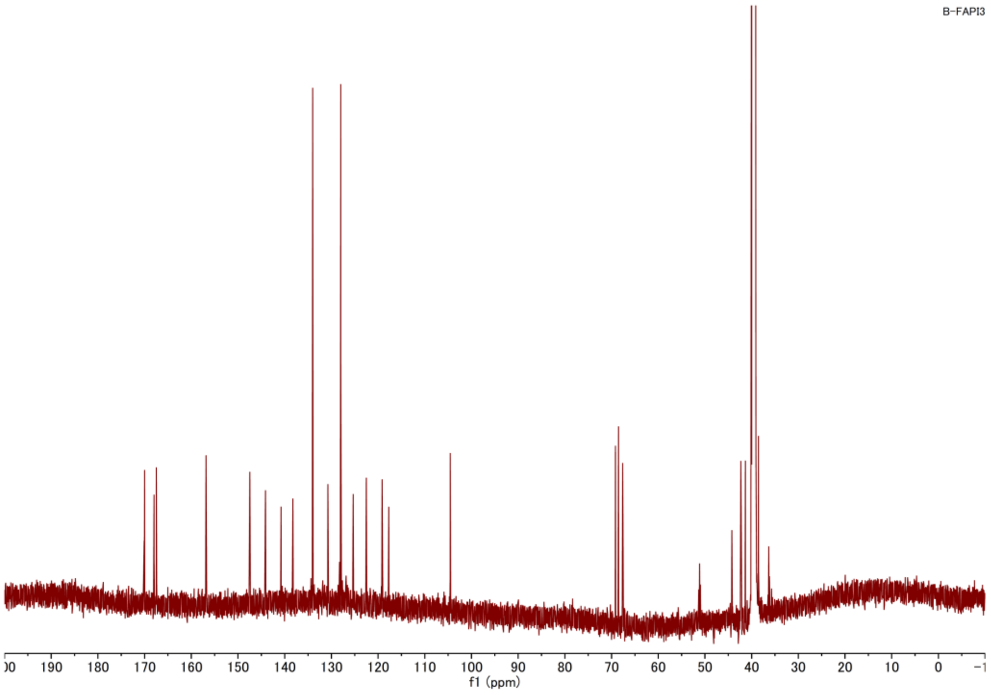

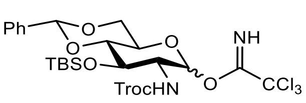
Ph
O
O
TBSO
O
NH
TrocHN
O
CCl3

S8

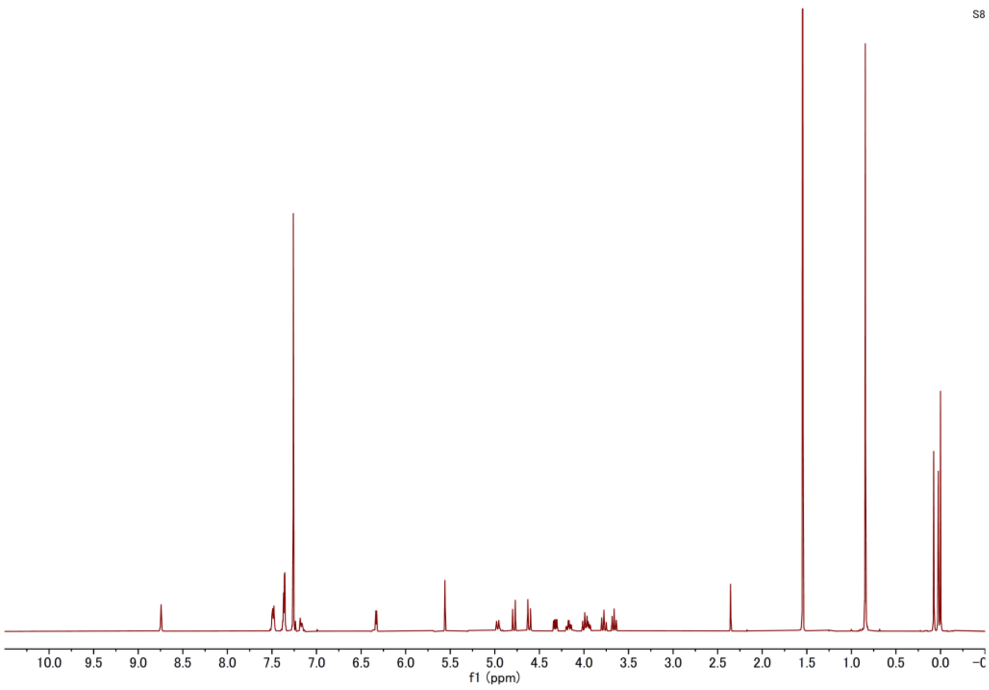
S8
10.0 9.5 9.0 8.5 8.0 7.5 7.0 6.5 6.0 5.5 5.0 4.5 4.0 3.5 3.0 2.5 2.0 1.5 1.0 0.5 0.0
f1 (ppm)

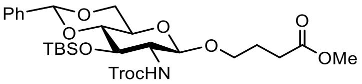

S9

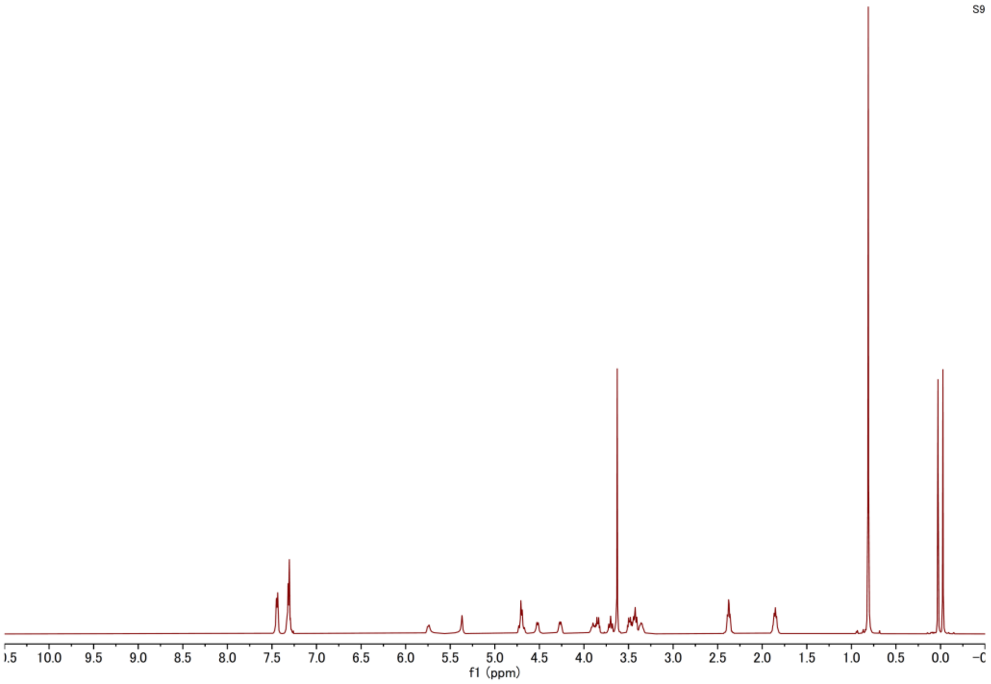

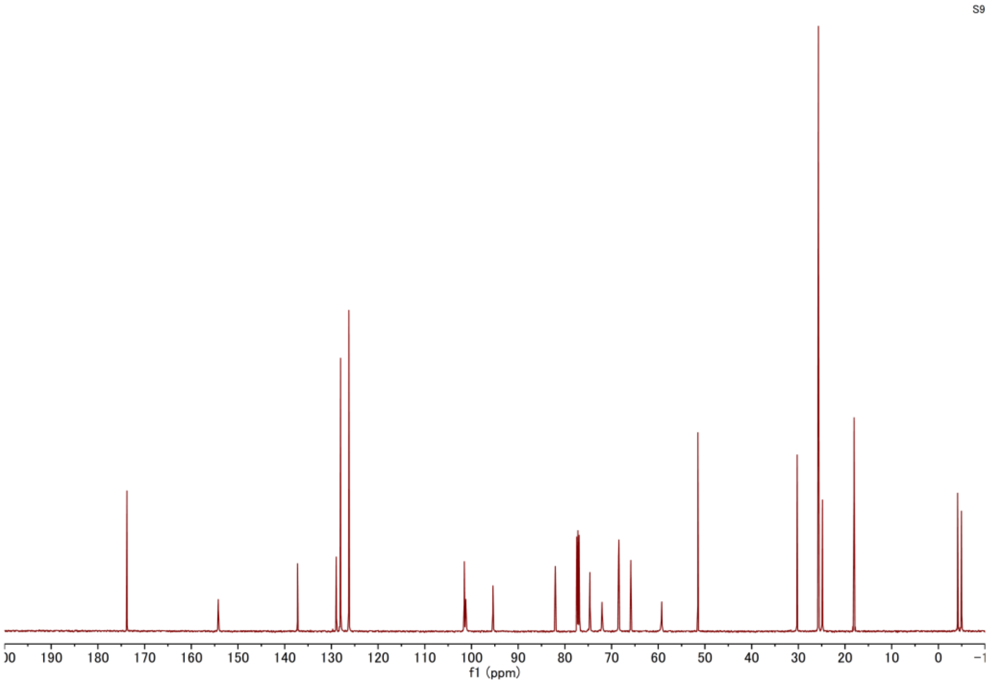

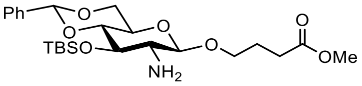

S10

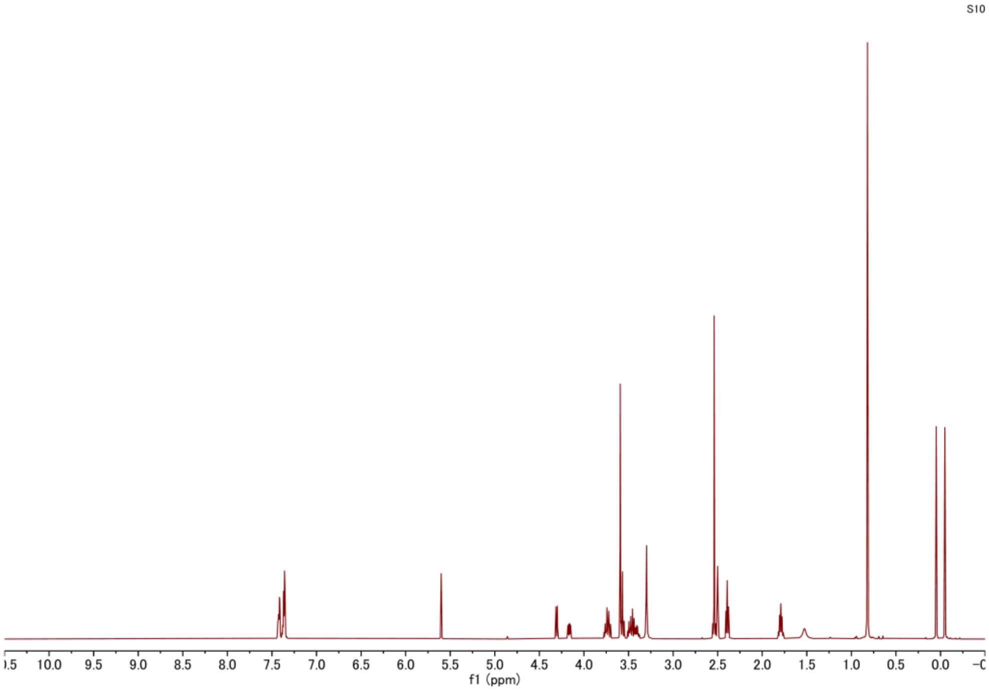

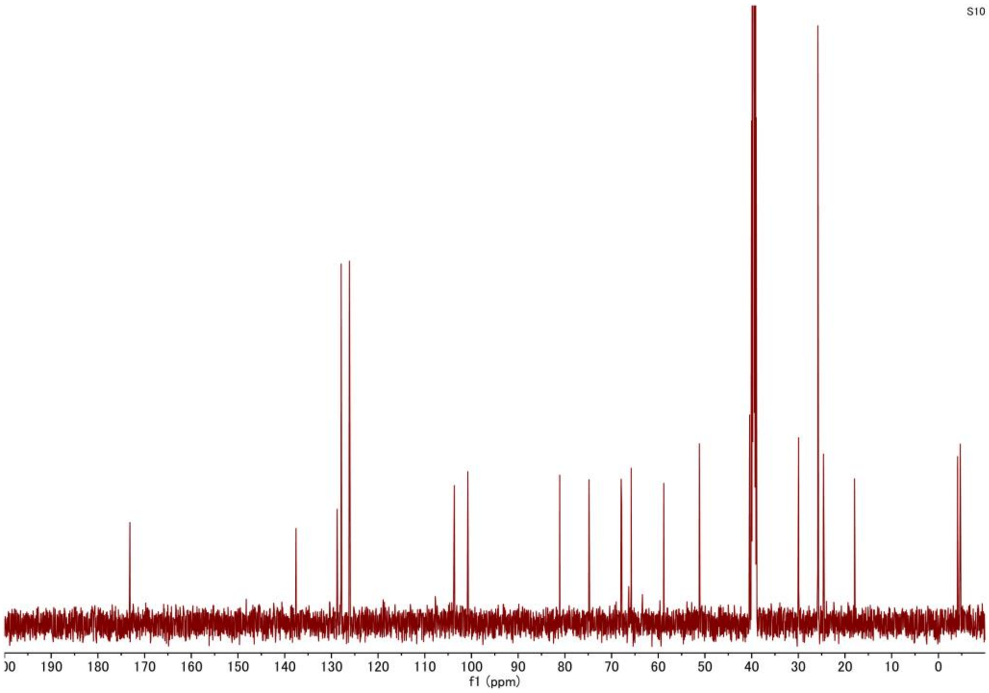

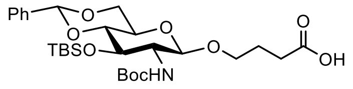
Ph
O
O
O
O
TBSO
BocHN
O
OH

S12

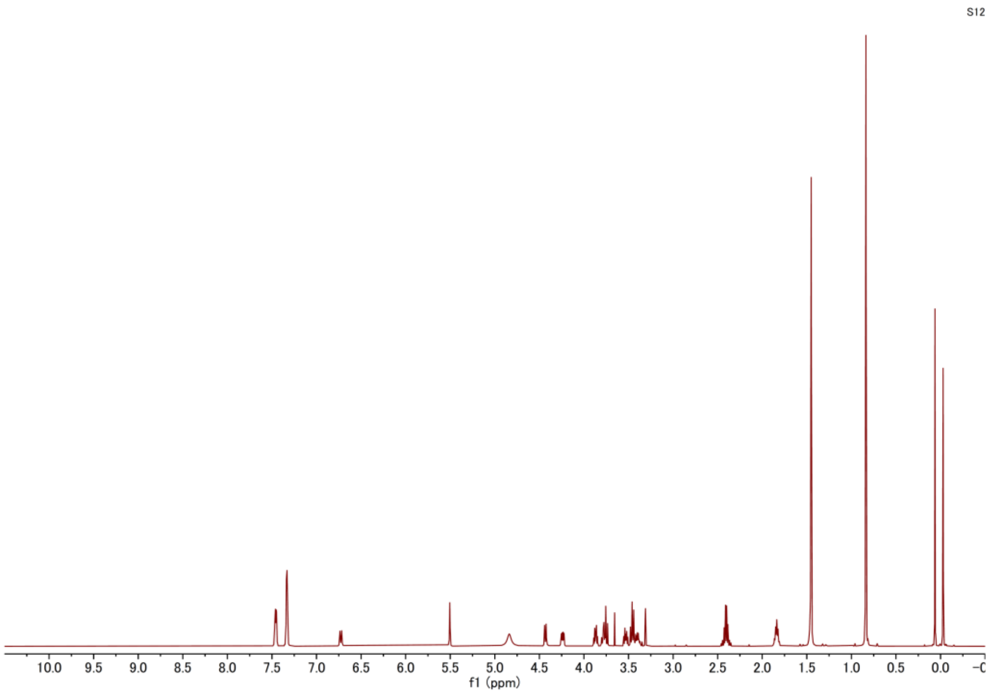
S12
10.0 9.5 9.0 8.5 8.0 7.5 7.0 6.5 6.0 5.5 5.0 4.5 4.0 3.5 3.0 2.5 2.0 1.5 1.0 0.5 0.0
f1 (ppm)

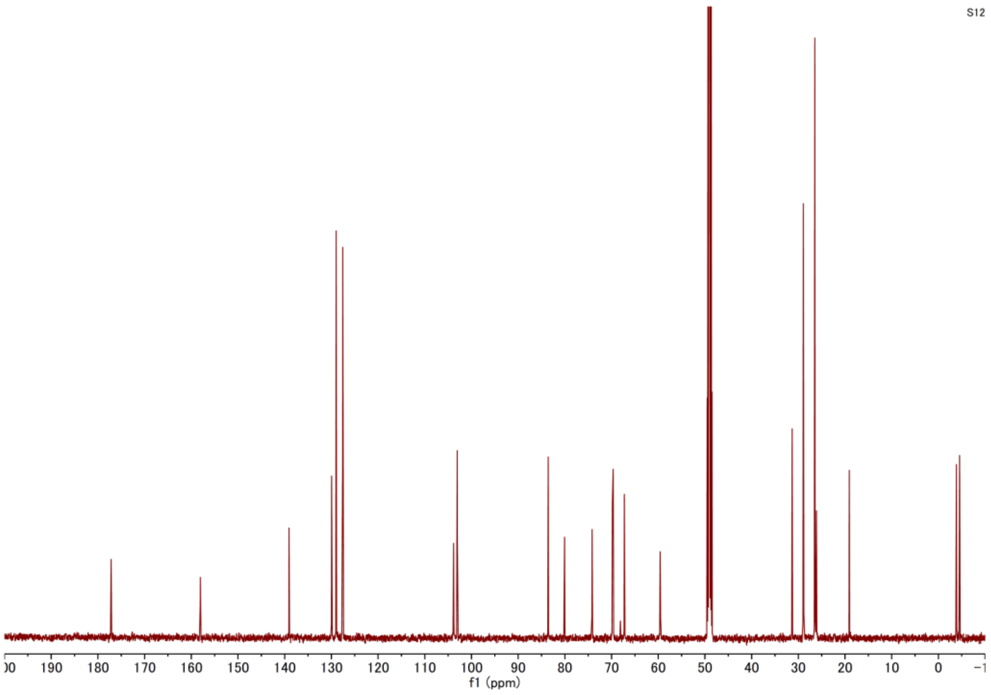
S12
190 180 170 160 150 140 130 120 110 100 90 80 70 60 50 40 30 20 10 0
f1 (ppm)

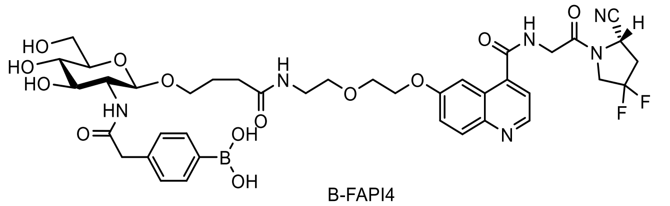
B-FAPI4

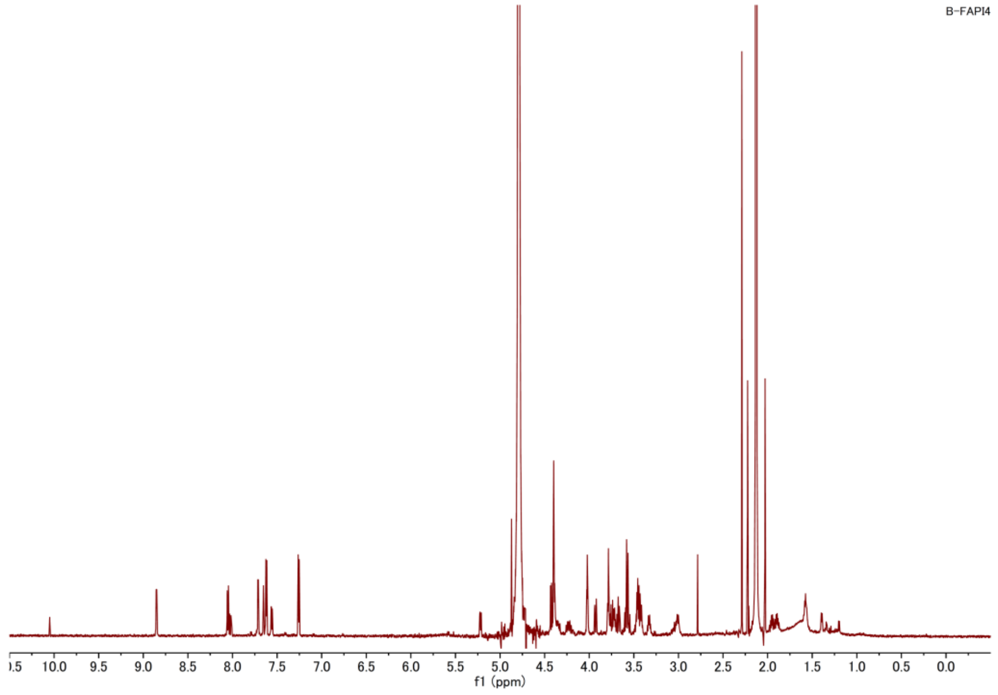

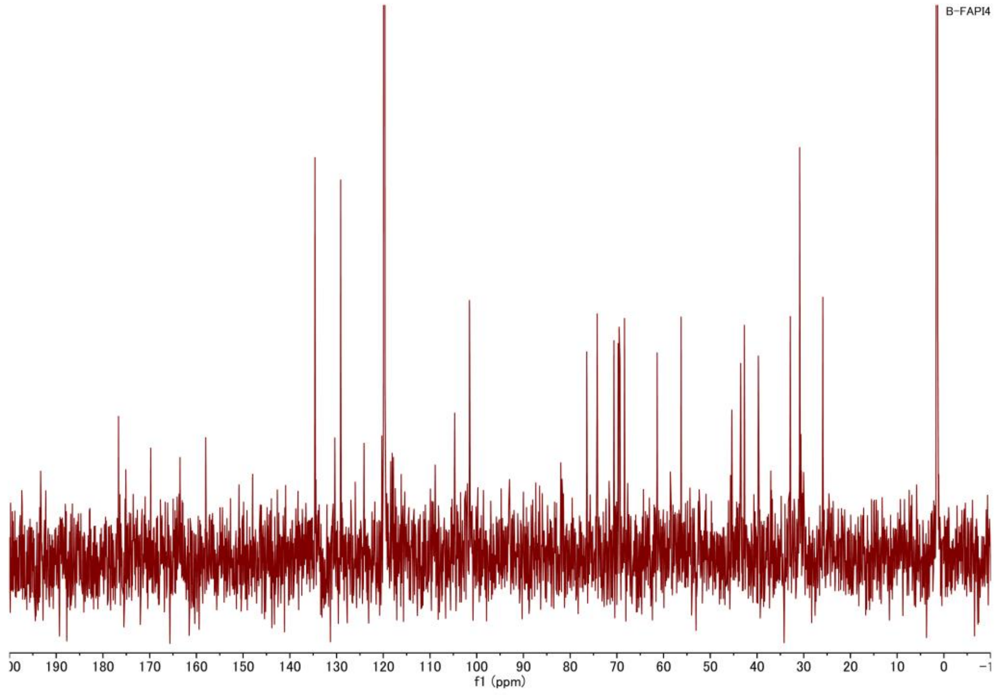

1-9. MS spectra

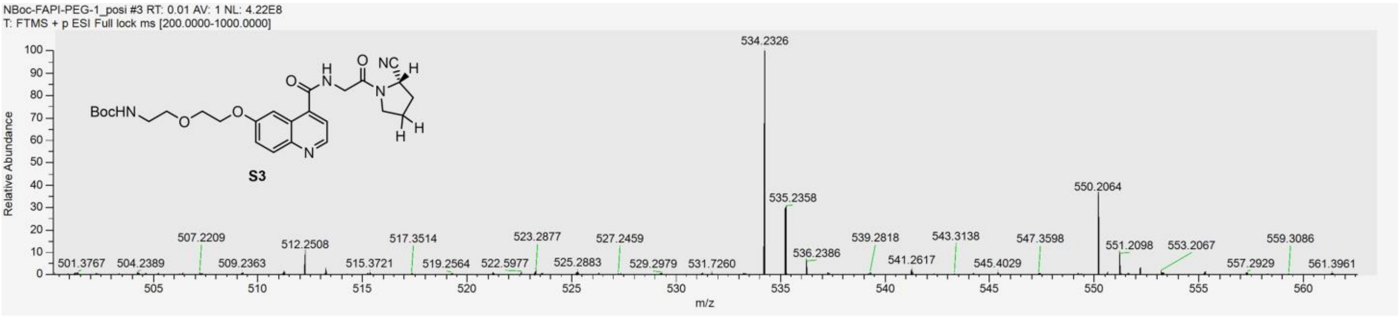
NBoc-FAPI-PEG-1_posi #3 RT: 0.01 AV: 1 NL: 4.22E8
T: FTMS + p ESI Full lock ms [200.0000-1000.0000]
Relative Abundance
m/z
534.2326
535.2358
550.2064
S3
BocHN
NC
H
N
O

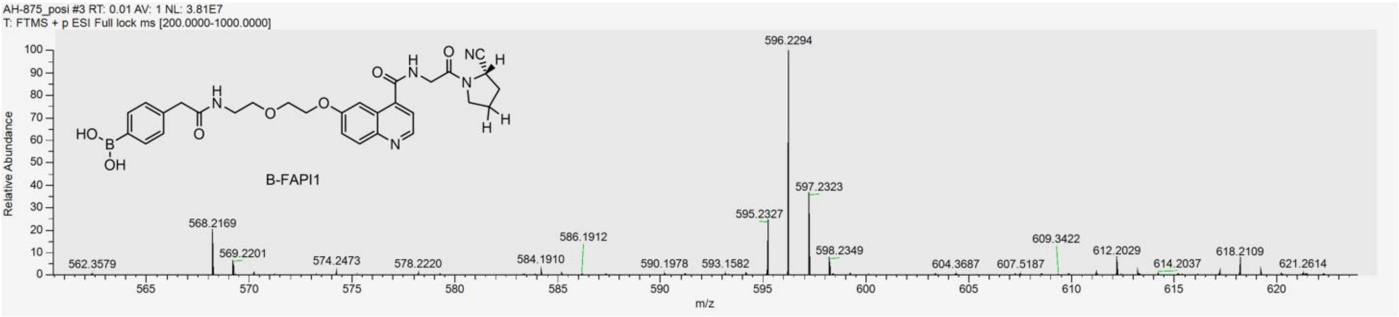
AH-875_posi #3 RT: 0.01 AV: 1 NL: 3.81E7
T: FTMS + p ESI Full lock ms [200.0000-1000.0000]
Relative Abundance
m/z
596.2294
597.2323
595.2327
568.2169
B-FAPI1
HO
B
OH

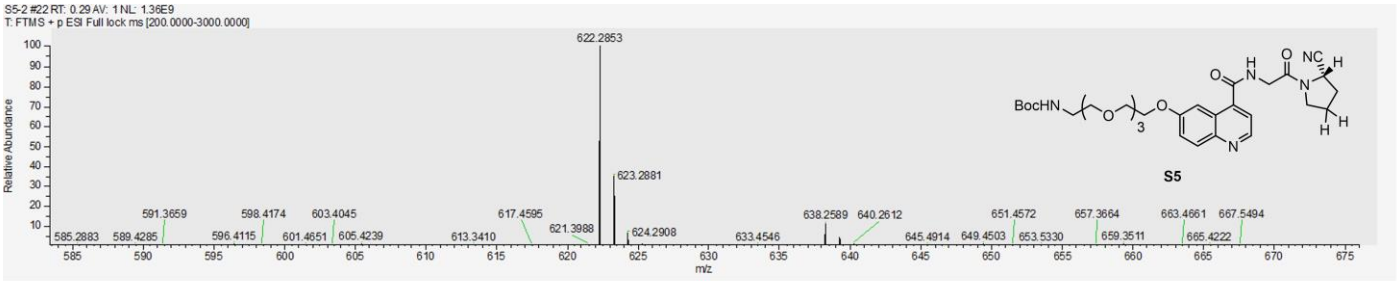
S5-2 #22 RT: 0.29 AV: 1 NL: 1.36E9
T: FTMS + p ESI Full lock ms [200.0000-3000.0000]
Relative Abundance
m/z
622.2853
623.2881
S5
BocHN
3

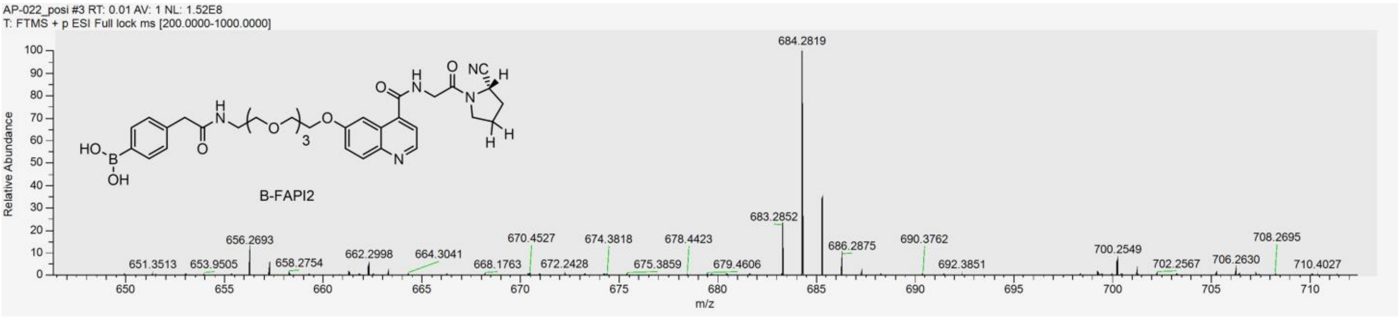
AP-022_posi #3 RT: 0.01 AV: 1 NL: 1.52E8
T: FTMS + p ESI Full lock ms [200.0000-1000.0000]
Relative Abundance
m/z
684.2819
683.2852
686.2875
B-FAPI2
HO
B
OH
3

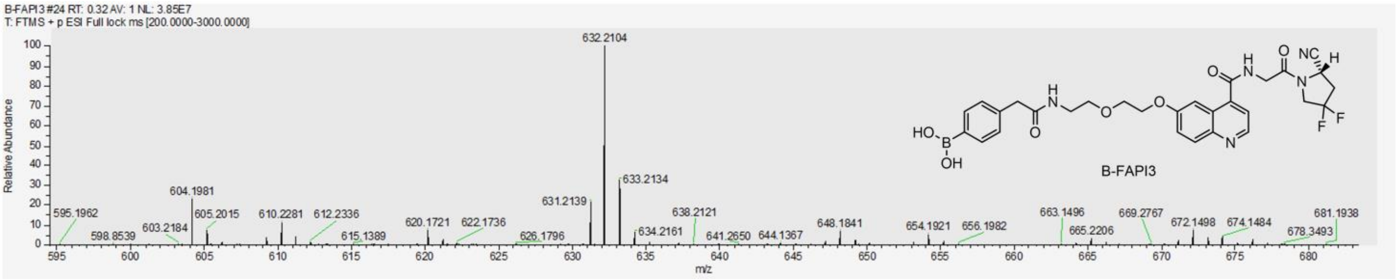
B-FAPI3 #24 RT: 0.32 AV: 1 NL: 3.85E7
T: FTMS + p ESI Full lock ms [200.0000-3000.0000]
Relative Abundance
m/z
632.2104
633.2134
631.2139
604.1981
B-FAPI3
HO
B
OH
F
F

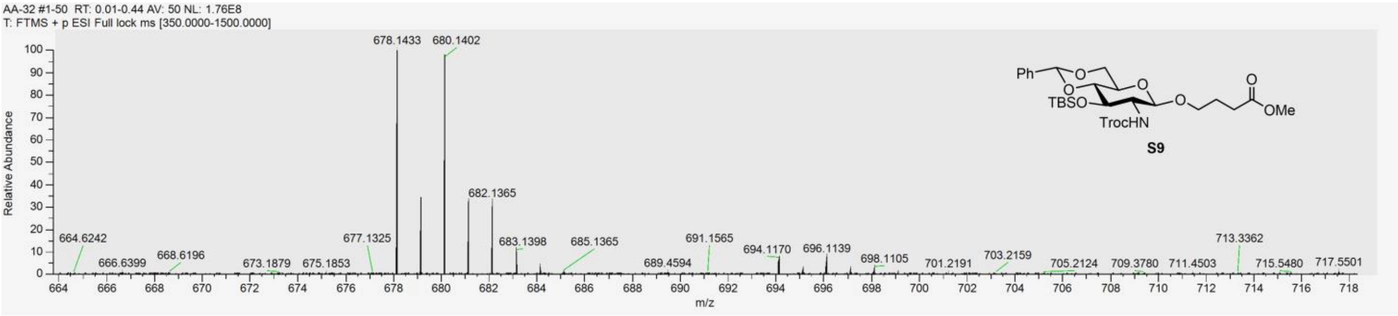
AA-32 #1-50 RT: 0.01-0.44 AV: 50 NL: 1.76E8
T: FTMS + p ESI Full lock ms [350.0000-1500.0000]
678.1433
680.1402
682.1365
Relative Abundance
m/z
S9

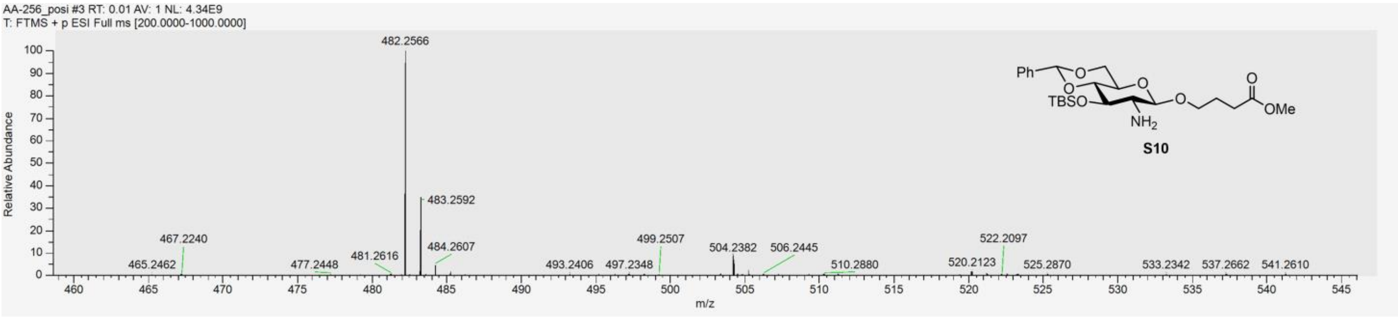
AA-256_posi #3 RT: 0.01 AV: 1 NL: 4.34E9
T: FTMS + p ESI Full ms [200.0000-1000.0000]
482.2566
483.2592
Relative Abundance
m/z
S10

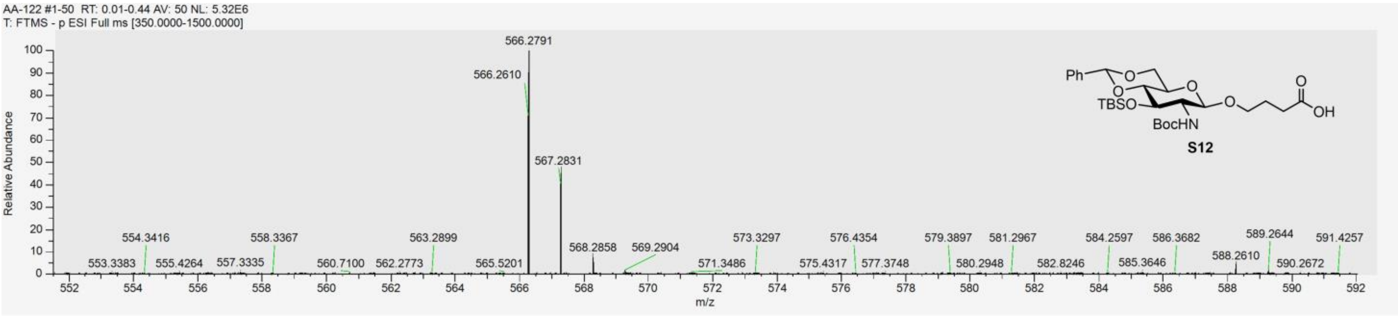
AA-122 #1-50 RT: 0.01-0.44 AV: 50 NL: 5.32E6
T: FTMS - p ESI Full ms [350.0000-1500.0000]
566.2791
566.2610
567.2831
Relative Abundance
m/z
S12

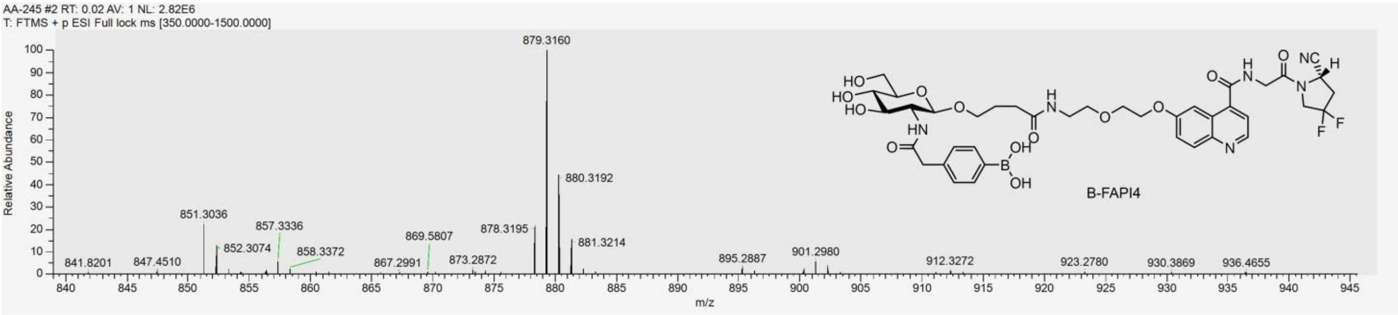
AA-245 #2 RT: 0.02 AV: 1 NL: 2.82E6
T: FTMS + p ESI Full lock ms [350.0000-1500.0000]
879.3160
880.3192
Relative Abundance
m/z
B-FAPI4

1-10. Synthetic procedure of ^{211}At-FAPI(s)

General procedure of ^{211}At-labeling

Overall, 0.1 % aqueous B-FAPI (10 μL), 7 % aqueous $NaHCO_3$ (10 μL), H_2O (90 μL), aqueous ^{211}At and 0.1 M aqueous KI (30 μL) were added to a polypropylene (PP) tube at room temperature. After being heated at 50 °C or 80 °C for 45 min, the reaction mixture was cooled down to room temperature. The radiochemical yield (RCY) was analyzed by TLC (CH_3CN/H_2O = 33:17). The TLC plate was exposed to an imaging plate, and then the imaging plate was scanned by an image analyzer. For purification, the reaction mixture was directly loaded to an Oasis HLB column, and the column was rinsed with H_2O (1.0 mL) and EtOH/H_2O (2:3) (500 μL), and then ^{211}At-FAPI1, ^{211}At-FAPI2, ^{211}At-FAPI3, ^{211}At-FAPI4 and ^{211}At-FAPI5 were eluted by EtOH (500 μL). After the purification, radiochemical purity (RCP) was analyzed using TLC.

Substrate	B-FAPI1	B-FAPI2	B-FAPI3	B-FAPI4	B-FAPI5
0.1 % aq. B-FAPI (μL)	10				
7 % aq. $NaHCO_3$ (μL)	10				
H_2O (μL)	90				
0.1M aq. KI (μL)	30				
aq. ^{211}At (μL)	14	2.1	19	19	10
Radioactivity (MBq)	0.28	0.66	0.62	0.60	1.01
Reaction time (min)	45				
Temperature (℃)	80		50		80
RCY (%)	100	99	45	15	90
RCP (%)	100	100	96	78	95

Table S1. Radiochemical yield (RCY) and Radiochemical Purity (RCP) of ^{211}At-FAPI1, ^{211}At-FAPI2, ^{211}At-FAPI3, ^{211}At-FAPI4 and ^{211}At-FAPI5.

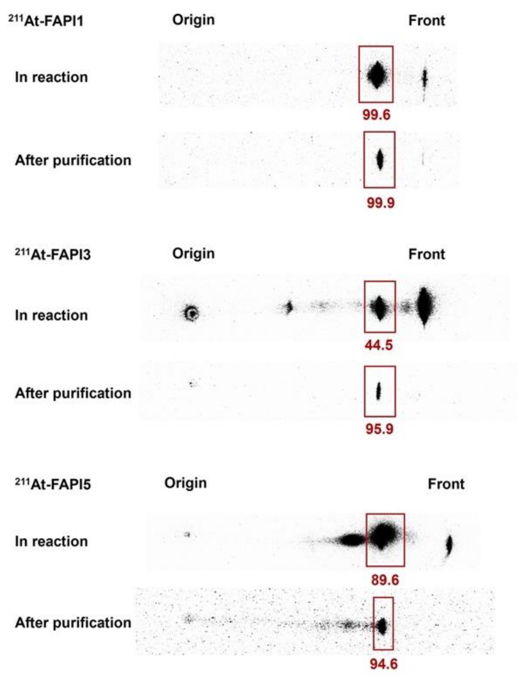

Figure S1. TLC analysis of ^{211}At-FAPI1, ^{211}At-FAPI2, ^{211}At-FAPI3, ^{211}At-FAPI4 and ^{211}At-FAPI5.

^{211}At-FAPI1			^{211}At-FAPI5		
No.	RCY (%)	RCP (%)	No.	RCY (%)	RCP (%)
1	98.5	99.5	1	89.6	90.9
2	97.8	96.4	2	81.4	75.0
3	98.0	94.7			
4	98.8	99.9			
5	99.6	99.9			
Average	98.5	98.1	Average	85.5	83.0
SD	0.7	2.4	SD	5.8	11.2

Table S2. Average ± SD of radiochemical yield (RCY) and Radiochemical Purity (RCP) of ^{211}At-FAPI1 (n = 5) and ^{211}At-FAPI5 (n = 2).

1-11. Synthetic procedure of ^{131}I-FAPI(s)

General procedure of ^{131}I-labeling

B-FAPI (1 mg), H_2O (80 μL), aqueous ^{131}I-NaI and 0.4 % aqueous *N*-Bromosuccinimide (NBS) (30 μL) were added to polypropylene (PP) tube at room temperature. After being heated at 80 °C for 30 minutes, the reaction mixture was cooled down to room temperature. The radiochemical yield (RCY) was analyzed by TLC (CH_3CN/H_2O = 33:17). The TLC plate was exposed to an imaging plate, and then the imaging plate was scanned by an image analyzer. After adding 4 % aqueous ascorbic acid (10 μL), the mixture was stirred for 15 min. For purification, the reaction mixture was directly loaded to an Oasis HLB column, and the column was rinsed with H_2O (1.0 mL) and EtOH/H_2O (2:3) (500 μL), and then ^{131}I-FAPI1 and ^{131}I-FAPI5 were eluted by EtOH (500 μL). After the purification, radiochemical purity (RCP) was analyzed via TLC.

Substrate	B-FAPI1	B-FAPI5
B-FAPI (mg)	1	
H_2O (μL)	80	
0.4 % aq. NBS (μL)	30	
aq. ^{131}I (μL)	28	30
Radioactivity (MBq)	6.76	6.77
Reaction time (min)	30	
Temperature (℃)	80	
RCY (%)	13	51
RCP (%)	91	99

Table S3. Radiochemical yield (RCY) of ^{131}I-FAPI1 and ^{131}I-FAPI5.

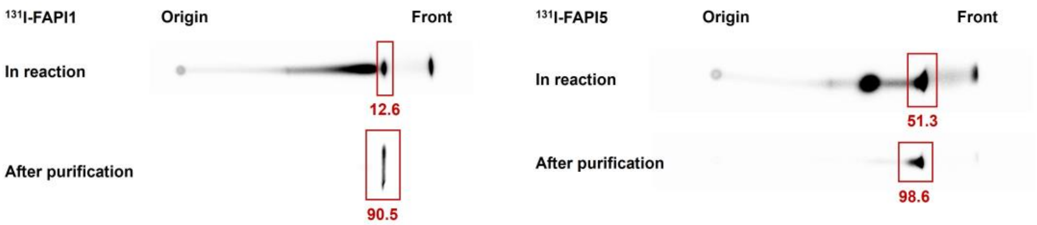

Figure S2. TLC analysis of ^{131}I-FAPI1 and ^{131}I-FAPI5

2. References

1. Hu, K.; Li, J.; Wang, L.; Huang, Y.; Li, L.; Ye, S.; Han, Y.; Huang, S.; Wu, H.; Su, J.; Tang, G. Preclinical evaluation and pilot clinical study of [^{18}F]AlF-labeled FAPI-tracer for PET imaging of cancer associated fibroblasts. Acta Pharm. Sinica B 2022, 12, 867–875. DOI: 10.1016/j.apsb.2021.09.032
2. Scholz, S. O.; Farney, E. P.; Kim, S.; Bates, D. M.; Yoon, T. P. Spin-Selective Generation of Triplet Nitrenes: Olefin Aziridination through Visible-Light Photosensitization of Azidoformates. Angew. Chem. Int. Ed. 2016, 55, 2239–2242. DOI: 10.1002/anie.201510868
3. Aso, A.; Kaneda-Nakashima, K.; Nabetani, H.; Kadonaga, Y.; Shirakami, Y.; Watabe, T.; Yoshiya, T.; Mochizuki, M.; Koshino, Y.; Ooe, K.; Kawakami, A.; Jinno, N.; Toyoshima, A.; Haba, H.; Wang, Y.; Cardinale, J.; Giesel, F. L.; Shimoyama, A.; Fukase, K. Substrate Study for Dihydroxyboryl Astatine Substitution Reaction with Fibroblast Activation Protein Inhibitor (FAPI). Chem. Lett. 2022, 51, 1091-1094. DOI: 10.1246/cl.220391
