# Supplementary material for: Evaluation of Astatine-211-Labeled Fibroblast Activation Protein Inhibitor (FAPI): Comparison of Different Linkers with Polyethylene Glycol and Piperazine
Source: Int J Mol Sci. 2023 May 12;24(10):8701. doi: 10.3390/ijms24108701 (PMC10218645; doi:10.3390/ijms24108701)
Supplement: Supplementary file 1 [file ijms-24-08701-s001.zip › ijms-2345109-supplementary S2.pdf]

International Journal of *Molecular Sciences*

MDPI

Evaluation of Astatine-211-Labeled Fibroblast Activation Protein Inhibitor (FAPI): Comparison of Different Linkers with Polyethylene Glycol and Piperazine

Ayaka Aso, Hinako Nabetani, Yoshifumi Matsuura, Yuichiro Kadonaga, Yoshifumi Shirakami, Tadashi Watabe*, Taku Yoshiya, Masayoshi Mochizuki, Kazuhiro Ooe, Atsuko Kawakami, Naoya Jinno, Atsushi Toyoshima, Hiromitsu Haba, Yang Wang, Jens Cardinale, Frederik L. Giesel, Atsushi Shimoyama, Kazuko Kaneda-Nakashima*, Koichi Fukase

Supporting Information

Establishment of FAPα over expression cell lines

Lentivirus vector was produced by the widely known protocol. pLenti-GIII-CMV-RFP-2A-Puro was transfected into 293T cells with two plasmids (pCAG-HIVgp as envelope and pCMV-VSV-G-RSV-Rev as virus protein) by according to HilyMax (DOJINDO LABORATORIES, Kumamoto, Japan) transfection protocol. 293T cells were kindly provided from RIKEN. Once produced, lentivirus was used for stable-cell line generation.

Infected A549 and HEK293 cells were selected under puromycin existing medium. After single cloning using RFP fluorescence as a marker, the cells were scaled up, and finally FAPα expression was confirmed with a flow cytometer.

Measurement of FAPα expression

Cells were dispersed by Trypsin-EDTA treatment, and washed three times by PBS(-). Counted and resuspended 1×10^5 cells/tube in 50 μL MACS buffer: 2mM EDTA(pH8.0) , 0.5 % Bovine Serum Albumin (BSA) in PBS(-). Cells were treated with FAPα antibody (FAB3715A, R&D Systems, Minneapolis, MN, USA) on ice for 30 minutes, and washed three times by PBS(-), and analyzed by Attune NxT flow cytometer. Over expression cell lines had high red fluorescence intensity and FAPα expression (**Supplementary Figure 1**).

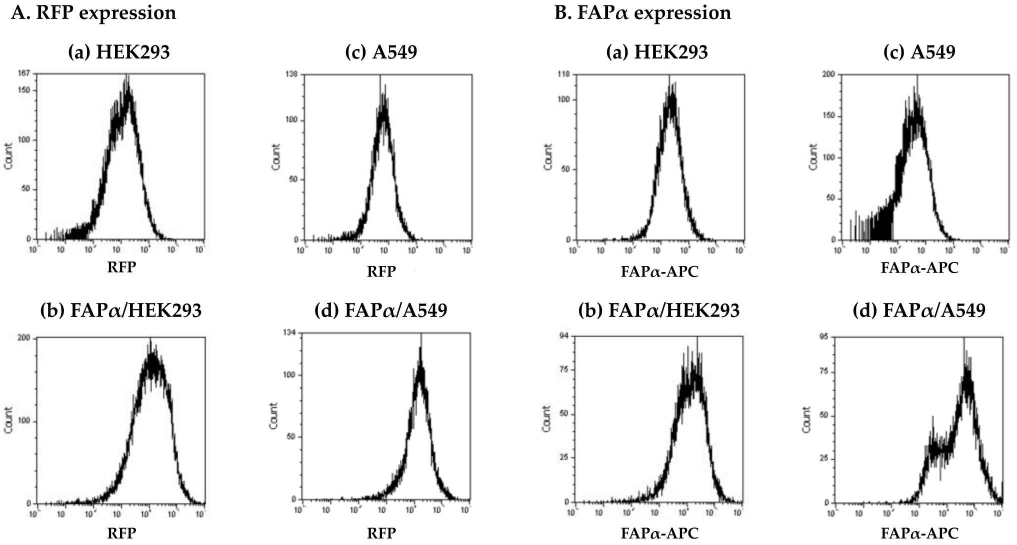

Supplementary Figure S1. Confirmation of expression levels of FAPα in established cultured cell lines. The vertical axis of the histogram indicates the number of cells, and the horizontal axis indicates the expression level of RFP (A) or FAPα (B).
